# Static and dynamic optimisation of fluid-filled responsive orthotic insoles

**DOI:** 10.1007/s10237-025-01935-w

**Published:** 2025-03-03

**Authors:** Dayna Cracknell, Mark Battley, Justin Fernandez, Maedeh Amirpour

**Affiliations:** Department of Engineering Science, The University of Auckland, Khyber Pass Road, Auckland, 1023 New Zealand

**Keywords:** Orthotic insoles, Solid–liquid composite, Additive manufacturing

## Abstract

This study was focused on developing an optimisation-based methodology to create customised solid–liquid composite (SLC) orthotic insoles. The goal was to reduce peak plantar pressures through gait through a dynamic numerical optimisation. A gait simulation was developed through a series of numerical models with increasing complexity. These models were validated against experimental analyses. The insole was designed based on numerical optimisation techniques that regionally tailored the insole with the aim to reduce temporal peak pressures. A prototype of the optimised insole was created using additive manufacturing and tested experimentally. The numerical gait simulation showed good correlation with experimental results. The largest differences are attributed to the bone geometry adopted from a previous study from a subject of different age, gender and size demographics. The optimisation process showed significant reductions in peak plantar pressures in the static peak pressures by approximately 8% and in the summation of dynamic peak pressures by 50%. Experimental validation confirmed the numerical predictions, highlighting the effectiveness of the optimised insole. The findings suggest that the optimised insoles can improve plantar pressure distributions and reduce peak pressures, making them a viable alternative to traditional orthotic insoles. Future research should focus on more accurate geometry for the numerical models and clinical trials.

## Introduction

Orthotic insoles are prescribed to treat discomfort and injuries of the foot that are often caused by high peak plantar pressures. Traditionally, the insole design is based on static pressure measurements, disregarding the changes in pressure distribution through gait (Landorf 2015; Yi et al. 2011; Smith et al. 2020). Peak pressures typically occur during gait when the body’s weight is concentrated on a small area on the foot (Landorf 2015), underscoring the importance of considering temporal pressure distribution in the design of effective insoles.

Recent advancements in orthotic insoles have been driven by additive manufacturing (Chhikara et al. 2022; Zuñiga et al. 2022; Ma et al. 2019; Anggoro et al. 2018; Peker et al. 2020). This technology offers economic benefits by minimising material waste and enabling cost-effective, individualised production (Attaran 2017; McDonald-Wharry et al. 2021; Amirpour and Battley 2022a, 2022b). Since the expiration of the FDM patent in 2009, which reduced 3D printer costs, research in this area has surged (Jemghili et al. 2020). Key studies have demonstrated the effectiveness of 3D-printed insoles in creating low-cost, personalised solutions and optimising insole geometry through numerical modelling (Scott et al. 2016; Telfer et al. 2017; Mannisi et al. 2019; Tsai and Chen 2018; Almeida et al. 2018; Tang et al. 2019; Ma et al. 2019; Chanda and Unnikrishnan 2019; Teixeira et al. 2021; Muir et al. 2022; Hudak et al. 2022; Channasanon et al. 2023; Shaulian et al. 2022; Kumar et al. 2023; Malki et al. 2024; Li et al. 2023; Geiger et al. 2023; Shaulian et al. 2023). Notable advances include smart insoles with embedded sensors (Alaimo et al. 2020; Gothard and Anton 2021; Samarentsis et al. 2022; Kim et al. 2022; Gothard et al. 2023; Willemstein et al. 2023; Macdonald et al. 2021; Najafi et al. 2017; Bus et al. 2011), and functionally graded designs that reduce peak plantar pressure by between 16% (Hudak et al. 2022) and 48% (Malki et al. 2024).

Responsive insoles adapt to external stimuli to improve foot strength, dynamic balance, and shock absorption. One subset of responsive insoles are fluid-filled insoles. Studies confirm that fluid-filled insoles have the ability to reduce peak pressures and absorb energy effectively (Miller et al. 2011; Anthony 2013; Kiper 2012; Ghassemi et al. 2015; Zhang et al. 2021; Alvarado-Rivera et al. 2022). However, an investigation into quantifying the improvement in terms of comfort of this design by Prader found that the fluid-filled insole performed poorly in comparison to the solid control (Prader 2018). It is speculated that this is due to the unfamiliarity of the orthotic rather than its performance (Hatton et al. 2015). Additionally, stability is a significant concern regarding the implementation of fluid-filled orthotics (Vanicek et al. 2009). Innovations include multi-material 3D-printed insoles and active responsiveness mechanisms like magnetorheological fluids, which further enhance impact absorption and pressure distribution.

A solid–liquid composite (SLC) for orthotic insoles combines tailored structural stiffness and graded permeability to provide a responsive functionality that passively redistributes pressure over the foot’s irregular topology, both statically and dynamically, to maximise user comfort (Cracknell et al. 2024a). The cellular structure is regionally tailored throughout the orthotic by varying unit cell size, thickness, and unit cell aspect ratios.

Changes in any of these geometric properties will alter the stiffness and nonlinearity in the mechanical response of the structure. Altering the geometry also tailors the permeability, affecting the resistance to flow and regional energy-absorbing capabilities. This research aims to develop an optimisation-based methodology to create a customised SLC orthotic insole.

## Methods

### Gait simulating numerical model

**Fig. 1:**
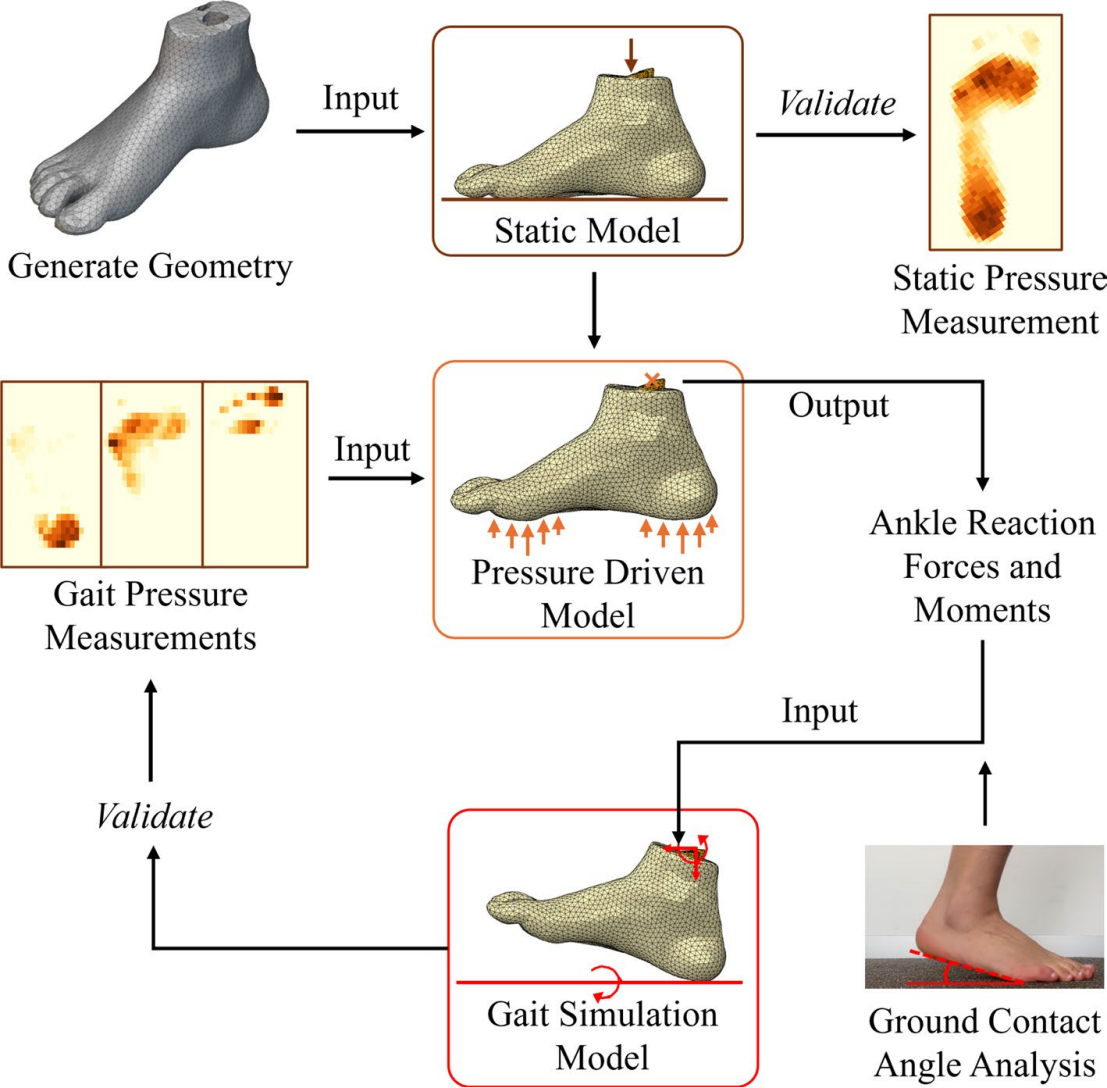
Process of developing a numerical model that simulates gait

The optimisation algorithm required a numerical model that can simulate gait and impose the plantar pressure experienced with walking. The primary aim was to replicate the plantar pressure distribution measured through experimental testing with a dynamic numerical simulation. The numerical modelling process was based on developing several subsequent models with increasing complexity in boundary conditions. First, a static model was developed to verify that the geometry was an appropriate representation of the subject’s anatomy. This would be completed by applying half of the subjects body weight, both in the model and experimentally, and comparing the resulting plantar pressure distributions. Then, a dynamic model was developed in two stages. The first was developed by applying experimental plantar pressure measurements to the geometry to obtain the reaction forces and moments at the proximal surface of the ankle. The final dynamic model took the resulting forces and moments from the previous model as boundary conditions to drive the loading of the foot during gait. According to Ozmanevra’s paper, this assumption is valid as they found that reaction forces remain relatively consistent despite variations in insole properties (Ozmanevra et al. 2018). In this model, the relative movement of the ground with respect to the foot was simulated based on video analysis. An overview of this process is shown in Fig. 1.

#### Geometry

The numerical model was based on the subject’s right foot. To create the model, the geometry of the foot had to be obtained. This was approached by generating a 3D scan of the foot from the ankle down to the plantar surface. A plaster cast of the subject’s foot was made to avoid movement of the foot while scanning, as the subject was scanning their own foot geometry. Although a direct scan would have been more efficient, it was not practical in this study.

**Fig. 2:**
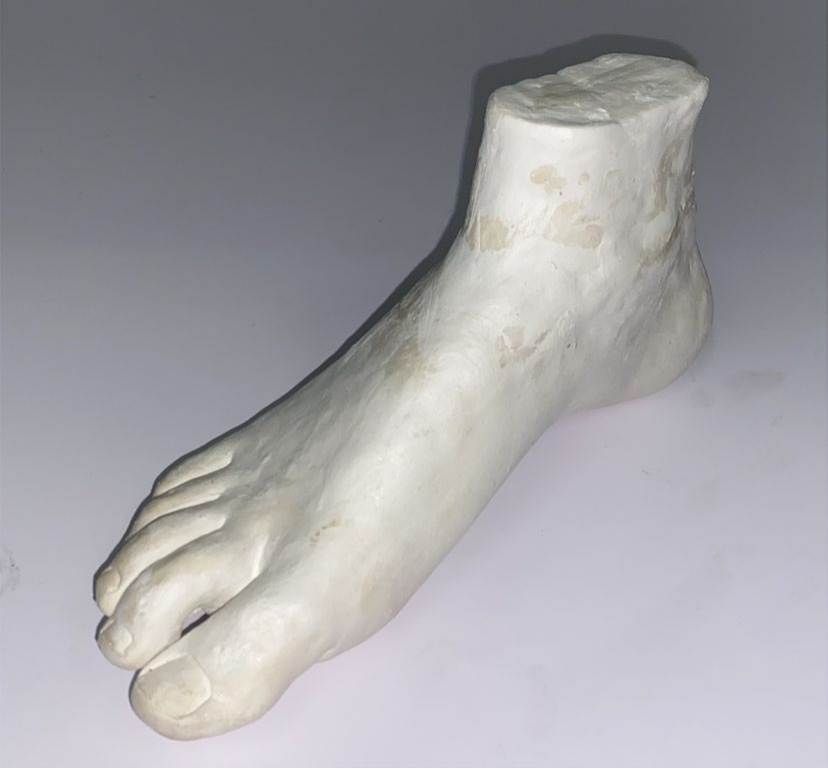
Plaster foot cast for 3D scanning

The negative foot mould was obtained using powdered Chromax Dental Alginate. The powder was mixed with water at a 5:8 ratio to a smooth consistency before immersing the foot in the mixture. As the mixture was very viscous, the subject could hold their foot in a posture that resembled a natural standing position. After the alginate had cured, the foot was removed. The positive mould was made by pouring Barne’s Casting Plaster into the cavity and waiting for it to set. The cast was removed by peeling off sections of the alginate to reveal the plaster underneath. The cast had imperfections from air pockets that were not filled with plaster. These holes were filled with Jovi Air Hardening Clay and buffed to blend with the plaster cast. An image of the final cast is provided in Fig. 2.

The foot cast was scanned using a Creaform 3D Scanner Version 8.1.7. The cast had to be scanned from multiple angles to obtain data on every surface, so the tracking dots were placed on a smooth surface and the cast was placed in the middle. The scan was taken from a top view and a bottom view by rotating the cast in two separate scans. Each scan was edited to remove unnecessary elements, such as the ground and surrounding objects. Then, the scans were aligned manually in VX elements post-processing, and their fit was optimised using an inbuilt surface-based method. The final scan was exported as an STL.

The final STL was not a perfect geometry as scanning between small gaps and detailed areas, particularly between the toes, was not possible, so the STL had to be repaired in mesh editing software. Altair’s Hypermesh version 2020.1 was chosen to fix the geometry. The first step involved manually repairing large holes by patching large sections with surface elements. A shrink-wrap was performed to patch all the small holes. A loose wrap was selected with an element size of $0.5\,\text {mm}$ to provide a smooth surface while capturing the scan’s details accurately. The hole patching option was selected with a minimum gap size of $20\,\text {mm}$ and a minimum hole size of $10\,\text {mm}$. The mesh was then assessed by checking the elements to ensure a closed volume had been created before being exported as an STL.

**Fig. 3:**
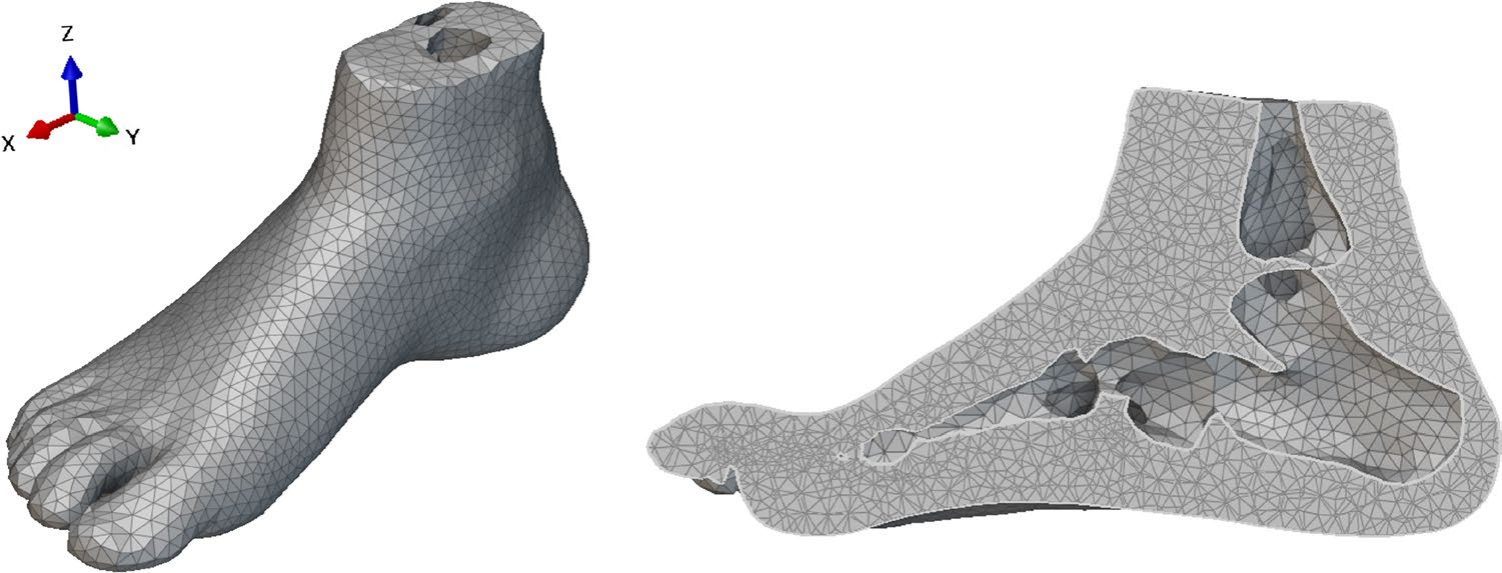
Post-processed foot mesh with bone cavity

The closed volume was then imported to nTopology. The purpose of this was to clean up the model and integrate cavities for the bone geometry. The STL mesh from Hypermesh was imported, and an implicit body was created based on the mesh. The bone structure taken from Kathirgamanathan’s model was then scaled to align with the subject’s foot (Kathirgamanathan et al. 2018). This required a 20% down scale in the Y and Z directions and a 30% scale down in the X direction. This modification is based on the assumption that modifying the bone structure to fit within the foot geometry is accurate enough to represent the bone structure in the subject’s foot for the purposes of this model. The bone structure was aligned with the bulk tissue. Its positioning was approximated manually, referring to the original MRI where the bone structure was taken from. After the alignment, a subtraction boolean was performed to create the cavity for the bones in the bulk soft tissue. The resulting implicit body was then converted to a mesh using an inbuilt robust tetrahedral mesh algorithm which optimises the quality of the mesh. An efficient mesh was a priority as it would create a faster and more stable model. The robust mesh was then converted to a FE mesh with linear tetrahedral elements with a hybrid formulation as the tissue would have a hyper-elastic constitutive model. The final mesh is shown in Fig. 3.

The model utilised a hyper-elastic constitutive material model for the soft tissue and a linear elastic model for the bones, as outlined in Table 1. A second-order polynomial constitutive model for soft tissue is commonly used to represent the bulk soft tissue (Chen et al. 2010; Kathirgamanathan et al. 2018) from in-vivo tests that Lemmon et al. conducted (Lemmon et al. 1997). However, the first-order Ogden model is also used in many cases (Erdemir et al. 2013; Isvilanonda et al. 2016; Chen et al. 2014). This first-order definition has been chosen for this model as the study by Erdemir showed that it has a strong correlation with a lumped soft tissue model compared to a layered soft tissue model (Erdemir et al. 2013). This constitutive model assumes any time-dependent tissue properties are negligible, particularly in later dynamic models. The elements in the soft tissue geometry were defined as linear tetrahedron with hybrid formulation and linear pressure (*C*3*D*4*H*) and the bones were linear tetrahedron (*C*3*D*4).

**Table 1:** Material models for each of the components

Component	Constitutive model	Material definition
Bones	Linear Elastic Kathirgamanathan et al. (2018)	$E = 7300\,\text{MPa}$, $\nu = 0.3$
Soft Tissue	$1^{st}$ Order Ogden Hyperelastic Erdemir et al. (2013)	$\mu _{1} = 0.0266$, $\alpha _{1} = 17.68$, $D_{1} = 0.523$
Ground	Analytically Rigid Kathirgamanathan et al. (2018)	$\infty$

#### Experimental pressure measurements

A comprehensive approach that combined experimental data collection and detailed numerical analysis was taken to simulate gait in a numerical model. First, experimental analyses were conducted on the subject to acquire their gait data. A preliminary standing test was performed on a Tekscan 5250 pressure mat to obtain a static plantar pressure distribution. Next, a walking test was carried out where the subject walked across the Tekscan pressure mat, capturing the entire gait cycle at a frequency of 50 Hz to ensure each stage was recorded with precision.

Calibration of the pressure sensor was optimised by using the subject’s body weight as a known parameter, which aligned the sensor for accurate readings during subsequent loading. Multiple walking tests were repeated to minimise any altered gait caused by aiming for a small target area on the mat. Once a natural gait pattern was captured, data from the test was used to identify each stage of the gait cycle based on known contact patterns from existing studies (Cicciu et al. 2024; Chen et al. 2001). A complementary video analysis, conducted at floor height, provided a detailed view of the gait from heel strike to toe-off. The contact angle was determined as the average line of the plantar surface relative to the ground.

#### Static model

The numerical modelling drew from the model established by Kathirgamanathan (Kathirgamanathan et al. 2018). The model simplifies the foot anatomy by treating soft tissues as a bulk material and omitting tendons and ligaments. Kathirgamanathan’s research shows that such simplifications do not significantly compromise the model’s accuracy (Kathirgamanathan et al. 2018).

**Fig. 4:**
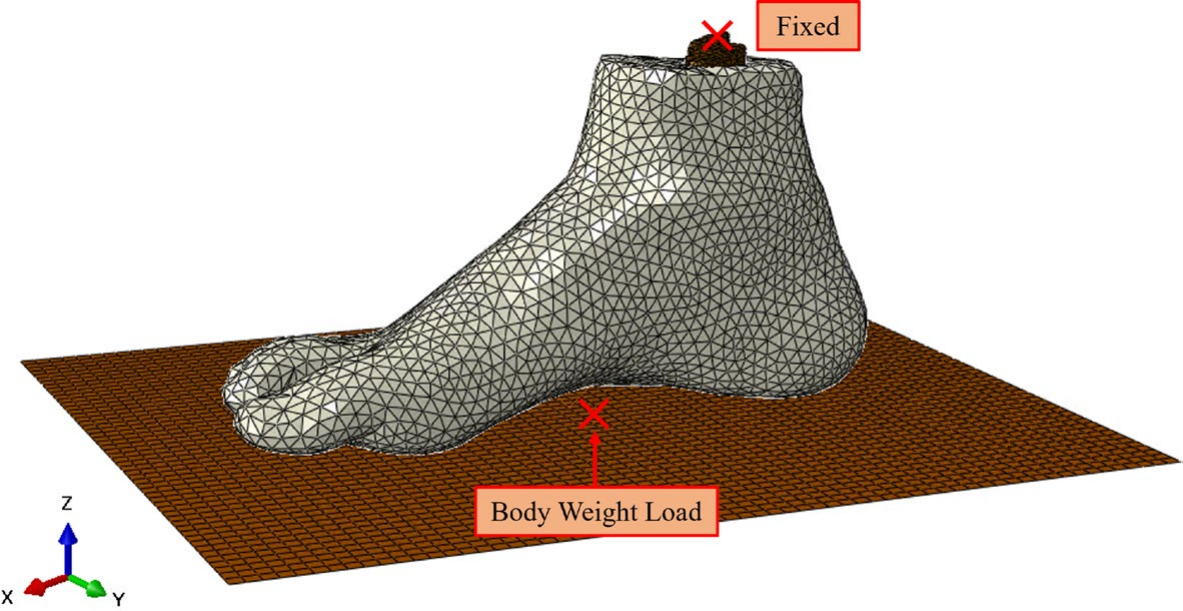
Static numerical model boundary conditions

The static foot model was created in ABAQUS/Standard, where all previous meshed components were imported. The assembly incorporates an analytically rigid ground plane aligned with the plantar surface of the foot. This initial static model aimed to simulate the subject standing to verify the geometry and material properties provided an accurate representation. All nodes on the proximal surface of the ankle bones were tied as a rigid surface to a reference point. This reference point was fully constrained in all degrees of freedom. Half of the subject’s body weight was applied to the analytically rigid ground’s reference point to simulate the loading conditions of the subject while standing. The numerical model is shown in Fig. 4.

The interaction between the ground and the plantar surface of the foot was modelled using a friction coefficient of 0.6. This friction definition was used as it is commonly adopted in literature (Hsu et al. 2008; Chen et al. 2003; Cheung and Zhang 2005, 2008).

A tie constraint was used to represent the connection between the bones and the soft tissue to simulate their bond. The ligaments and cartilage were excluded and instead lumped into the soft tissue. Each segment of bone was not connected and could therefore move with respect to each other under reasonable forces. An implicit static analysis was conducted with a gradually ramped load to simulate the body weight loading condition.

#### Dynamic gait model

In the first dynamic foot model that is driven by plantar pressures obtained experimentally, the overall assembly remained consistent with the static model, except for removing the analytically rigid surface plane representing the ground. Six steps were created sequentially to represent distinct loading stages of the gait cycle: heel strike, foot flat, mid-stance, terminal stance, heel off, and toe off.

Loading conditions for each stage were derived from an experimental gait analysis that captured pressure maps at each phase. The subject walked across a Tekscan pressure mat, and approximate pressure distributions at each stage were extracted and exported as x-y coordinates. These pressure maps were then implemented as analytical fields in ABAQUS/Standard. A coordinate system was created where the origin aligned with the position of the corner of the pressure mat, which properly aligned the field to the correct positions. This ensured that the X and axes aligned with the pressure map’s axes, and the Z-axis was perpendicular to the ground. Each node on the plantar surface was aligned with a pressure value according to its coordinates. The load was applied through a pressure load, with the plantar surface as the loading region.

**Fig. 5:**
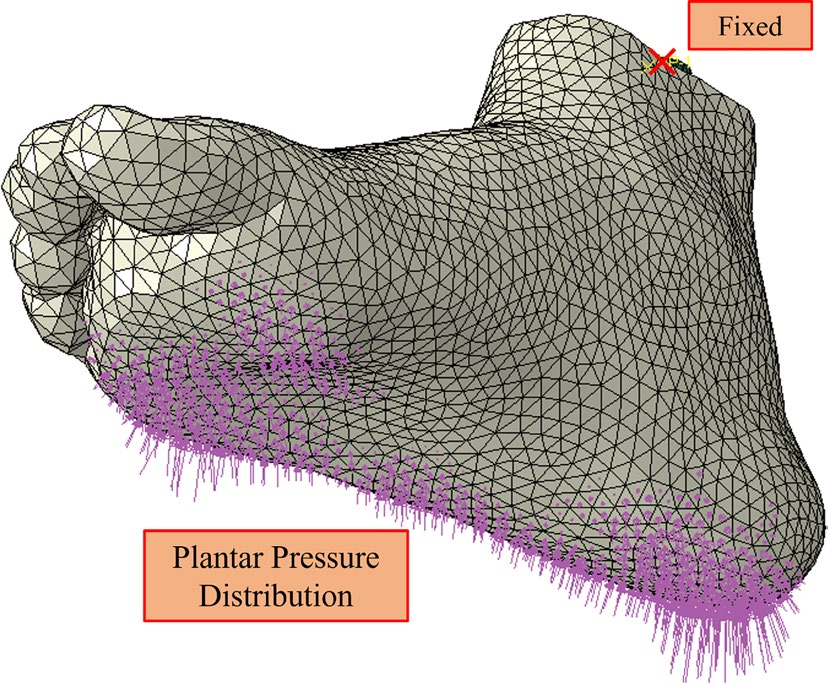
Plantar pressure-driven numerical model boundary conditions

Output data consisted of reaction forces and moments extracted from the top of the ankle bone, providing insight into the magnitude of the forces and moments experienced throughout the gait cycle. This data would then be implemented in the following dynamic model that simulated gait. Figure 5 shows the assembly and loading for the flatfoot step in this model.

The final gait model’s driving factors include the reaction forces at the proximal surface of the ankle found in the previous model and the contact angle between the ground and the plantar surface of the foot. The resulting forces from the pressure-driven dynamic model were used as inputs and applied to the proximal end of the ankle bone at the same reference point where the forces were measured. The contact between the foot and the ground was the same as applied in the static model with a frictional penalty coefficient of 0.6.

**Fig. 6:**
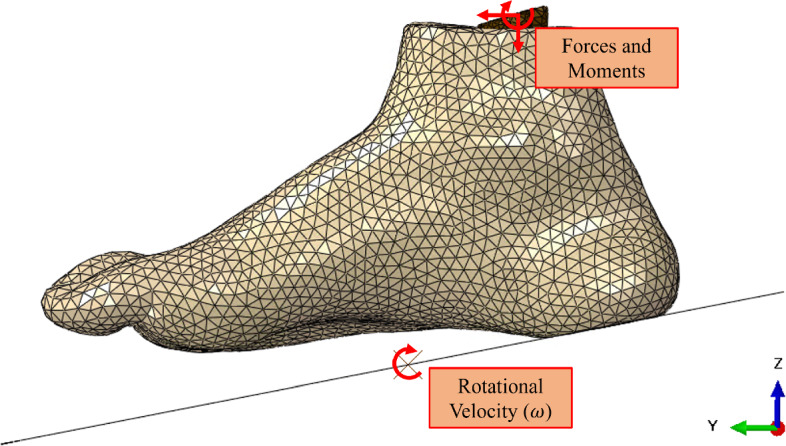
Gait driven numerical model boundary conditions

The ground was fixed in all directions aside from rotationally about the *X*-axis, which was defined as a constant rotational velocity in each step. These values were found experimentally where the subject was filmed at ground level while walking with natural gait. The video was taken at 60 frames per second to ensure each stage of gait was captured. This footage was post-processed by taking frames from each stance in the contact period of the gait cycle. These images were then imported into Adobe Illustrator 2023, where approximate lines were drawn along the ground and averaged along the plantar surface. The angle difference between these two lines was taken as the contact angle. The rotational velocities were calculated using Eq. 1. The application of these boundary conditions is illustrated in Fig. 6.

1 $$\begin{aligned} \omega = \frac{\theta _{\text {step}} - \theta _{\text {step} - 1}}{t_{\text {step}} - t_{\text {step} - 1}} \end{aligned}$$

### Optimisation algorithm for insole design

A gradient-based optimisation method has been developed for the design of a customised SLC orthotic insole. A total contact insole design was created based on the foot model. This geometry was implemented in the static numerical model and the model that simulates gait, before being run through an optimisation algorithm that minimises the peak plantar pressure by regionally adjusting material properties.

#### Dynamic model with insole

**Fig. 7:**
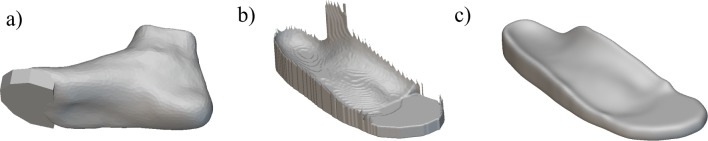
Development of the custom orthotic. **a** modified foot geometry, **b** manufacturing support voxel grid, and **c** smoothed final insole

Creating a custom orthotic insole for the subject was done on nTopology. The foot geometry discussed in the previous section was imported. An extruded polygon was modelled from the planar protection of the toes. This would ignore the contours of the toes when creating the orthotic, creating a flat tip as shown in Fig. 7a. This shape is generally used in orthotic insoles as the toes undergo a large amount of movement while moving, and they have a large role in an individual’s gait patterns. The insole was modelled based on a total contact design to aid in reducing peak plantar pressures. Contouring the insole to the topology of the plantar surface was done using a manufacturing support voxel grid analysis. This would extrude regions from a reference plane to the plantar surface. The reference plane was set to approximately $10\,\text {mm}$ below the peaked regions on the foot. The feature size was set to $1\,\text {mm}$, and the overhang angle was set to approximately 170 degrees. This provided a detailed replication of the plantar surface, as shown in Fig. 7b. This geometry was then post-processed with two rounds of smoothing operations to create the final insole shown in Fig. 7c.

The homogenised orthotic insole was added to the assembly for the gait simulation numerical model discussed in the previous model. It was aligned with the plantar surface of the foot geometry. The interaction between the foot and the insole was defined as a tangential penalty with a friction coefficient of 0.6, with no separation allowed after contact to keep the insole attached to the foot.

The material properties of the insole would vary with the optimisation algorithm, but they were initialised with values that represented the middle of the bounds (1.5 mm wall thickness, 7.5 mm cell size and 1 : 1 aspect ratio). Notably, the effect of viscoelasticity of the solid lattice has been omitted in this simulation as it has a small effect in comparison to the rate-dependent fluid effects, as outlined in previous work (Cracknell 2024, Cracknell et al. 2024a, b). In summary, the SLC insole uses hyperfoam and permeability constitutive material models to capture the behaviour of the lattice structure and the fluid.

**Fig. 8:**
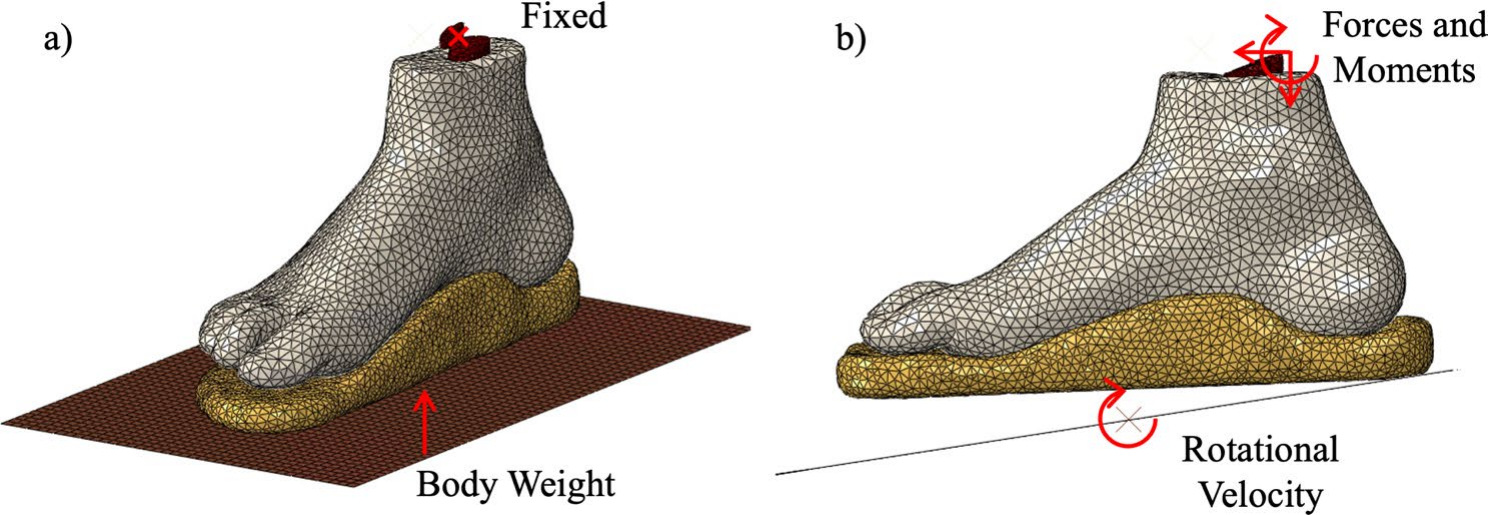
Numerical models with the insole implemented in the assembly. a) static model, and b) dynamic model

All of the solvers for each step were replaced with a Soils solver so it can analyse the fluid behaviour inside of the insole. This approach is outlined in previous work (Cracknell et al. 2024a). The elements for the insole were defined as a linear tetrahedron with coupled displacement-pore pressure (*C*3*D*4*P*). An image of the final assembly is shown in Fig. 8.

#### Optimisation algorithm

The optimisation model was used for static and dynamic analyses to compare the resulting geometry for both cases. This would show what effect the dynamic optimisation had on the results.

**Fig. 9:**
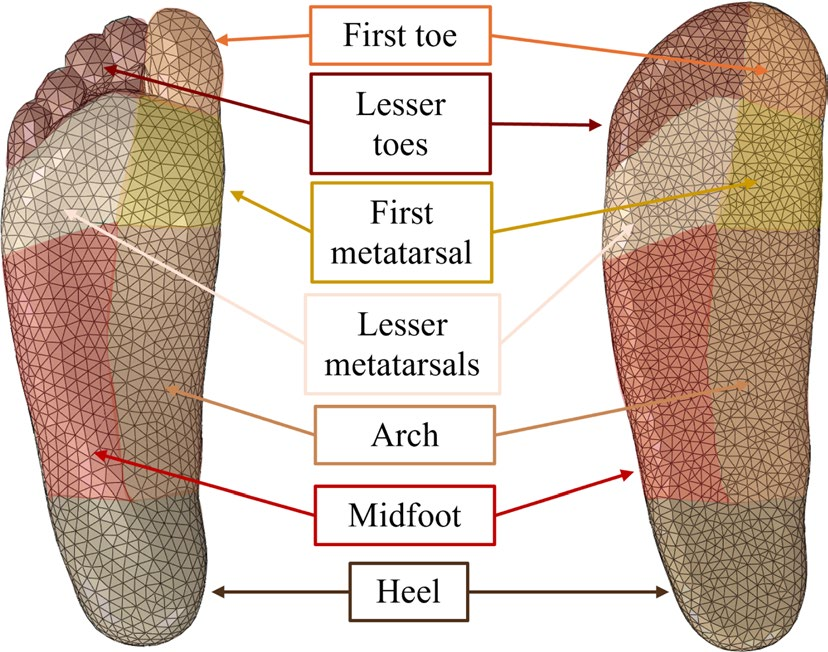
Defined regions on the insole and their respective area on the foot

The insole was split into seven regions; heel, arch, midfoot, first metatarsal, lesser metatarsals, first toe, and lesser toes. These regions are indicated in Fig. 9.

In each region, the geometric parameters are iteratively adjusted within specified bounds in a computational model. The objective of this iterative process is to minimise the peak plantar pressures. This will inevitably cause the pressure distribution on the plantar surface to become more even, offloading high pressure regions by adding more support into lower pressure regions. The model computes the plantar pressure for various geometric parameter assignments at each iteration, looking at a positive and negative step in each parameter. The parameters resulting in the lower peak plantar pressure indicate the direction of the steepest gradient, which informs subsequent adjustments. This iterative process continues until the variance in maximum stress is within a predefined tolerance. The objective function (*F*) for the algorithm is provided in equation 2, where *N* is the number of elements and $\bar{\sigma }$ is the mean stress.

2 $$\begin{aligned} F = \sqrt{\frac{1}{N} \sum _{i=1}^{N} (\sigma _i - \bar{\sigma })^2} \end{aligned}$$

Manufacturability considerations are embedded in the algorithm, which imposes bounds on the geometric coefficients to prevent the creation of structures that are either too thin for printing or too dense for efficient post-processing, such as excess resin removal. Experimental experience shows that wall thicknesses less than 1 mm thick tend to break and degrade the properties of the sample quickly. Wall thicknesses more than 2 mm for a cell size less than 7.5 mm tend to create voids to small to effectively post-process the sample with cleaning. Further limits were placed only to include samples that have been evaluated based on structures that can replicate the range of material properties found in traditional orthotic foams, as discussed in previous work (Cracknell 2024).

In this process, only alterations in the horizontal aspect ratios will be explored. The reason for this is multifaceted; the horizontal aspect ratio has a significantly larger effect on permeability in the primary direction of flow (parallel to the plantar surface). Additionally, an increase in the vertical aspect ratio is presumed to have undesirable effects as the buckling behaviour is more extreme. Omitting this additional geometric parameter will significantly improve computational efficiency.

The first stage of the algorithm includes the setup of constants in the script. It defines the Abaqus model, along with the number of regions that will be included in the optimisation process. The predefined material properties were implemented in a dictionary where the key was the geometric values (thickness, cell size and horizontal aspect ratio), and the value was their corresponding material properties (hyperfoam constants and permeability values).

The initialisation also defines the initial guesses, bounds, and default step size for each of the geometric values. The initial guess for all regions was defined as the middle value between the bounds. The bounds were imposed to ensure the optimisation stays within feasible and manufacturable limits.

- *Objective function update *(*obj_update*): This function took the lattice parameters and the new job name as inputs and ran the model with the current parameters set for the iteration. After running, it extracted the maximum stress from a predefined set of elements that make up the plantar surface of the foot. It also checked if the model ran until completion. If the model failed, the maximum stress was set to an unrealistically high value (100 MPa), so it will not be considered a viable option in the next iteration. This was based on the assumption that the material properties were not fit to withstand the loading conditions appropriately. This was seen when developing the model, where excessive distortion was a common reason a job was not completed. The resultant object function is returned from this function.
- *Sensitivity direction* (*obj_dir*): This function took the iteration’s initial set of parameters and the resulting objective function as inputs. It changes each parameter by a step value and evaluates the changes in the objective function. It outputs a normalised unit vector indicating the sensitivities of each material parameter in each region on the objective function.
- *Step size determination* (*find_step_size*): The inputs of this function include the objective function of the starting position, the initial parameters and the normalised sensitivity vector. This function iterates over different step factors, adjusting the parameters and evaluating the new objective value. It checks if the new parameters are within bounds and either exist in the predefined properties or require interpolation. The output of this function is a vector representing the best step size for each parameter to minimise the objective function.

The algorithm first runs the simulation with the initial parameters provided using the objective update function. This returns the maximum stress found in the output. Then, the main algorithm loop begins.

**Fig. 10:**
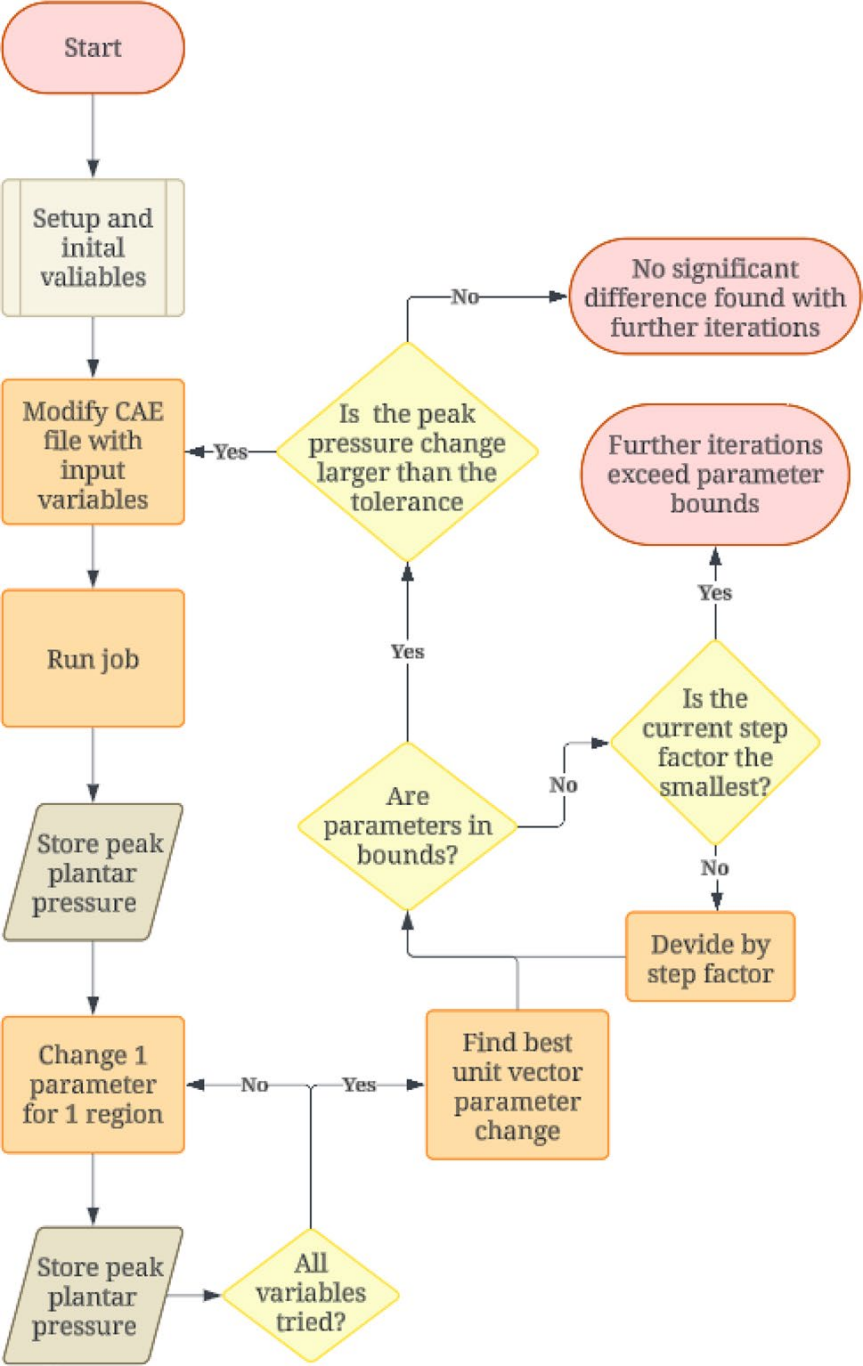
Flowchart representing the optimisation algorithm

The main interactions occur in a while loop that ensures the number of iterations does not exceed the maximum allowable defined in the initial setup. The function that determines the sensitivity directions (*obj_dir*) is called to find how a small change in each of the parameters in each region affects the objective function. If this function returns all zeros, the optimisation loop will stop, indicating no significant difference was achieved by changing any of the parameters. Otherwise, the step size determination function (*find_step_size*) is called to find the step size within the bounds that will minimise the objective function. The algorithm will stop if no step size can decrease the object function or if further iterations exceed the parameter bounds. After these checks are completed, the iteration number, objective function value, and region specific parameters are printed to a file and stored for the next iteration to begin. The algorithm will keep running until one of the checks breaks the loop. Figure 10 shows an overview of the algorithm.

### Insole performance

#### Insole geometry

The geometry for the final insole designs was created in nTopology version 4.10.2. The regionised geometry is created using a series of scalar point maps representing an aspect of the unit cell geometry; unit cell size in the X direction, Y direction, Z direction and the wall thickness. These were extracted from the results of the optimisation process.

**Fig. 11:**
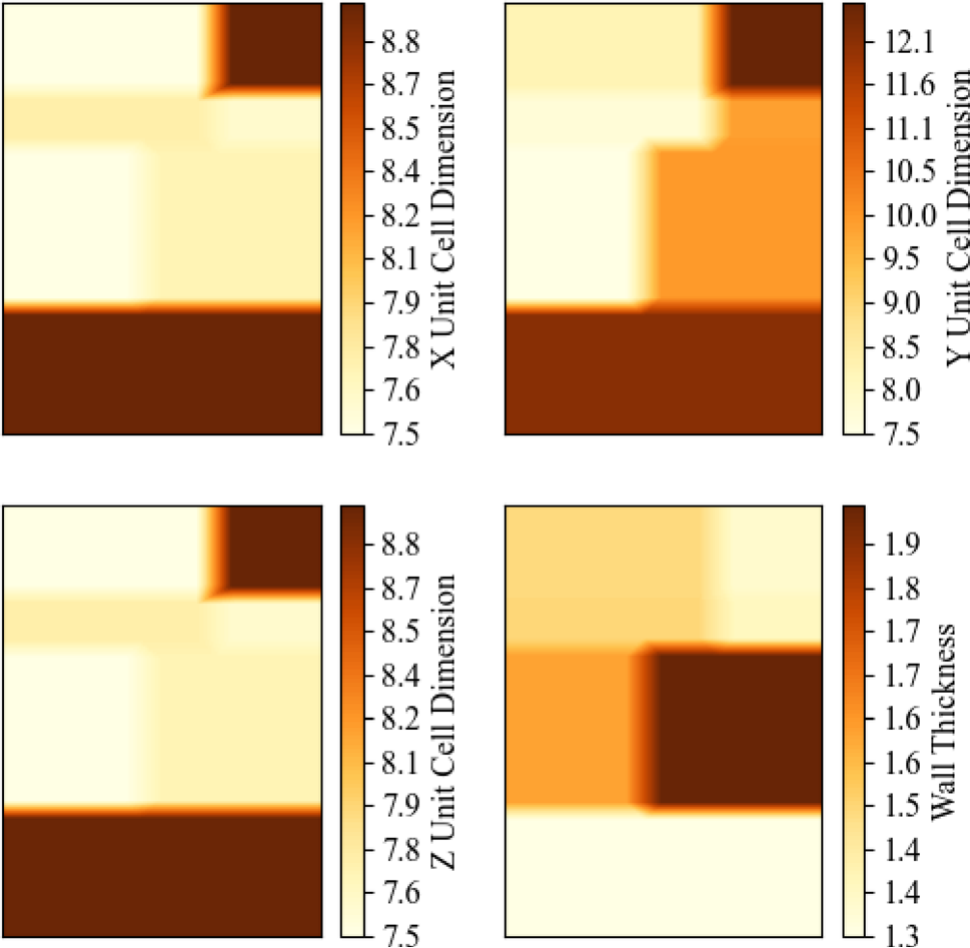
Spacial variations in the geometric controls of the lattice properties

To avoid harsh stiffness gradients in the insole, gradients at the boundaries between the regions were created. These gradients were created with a linear interpolation with 10 mm boundaries. The colour maps representing the values in each region and their respective boundary gradients are shown in Fig. 11. These spacial controls were imported into nTopology as scalar point maps. The point maps are converted to continuous fields.

The field values are translated to geometric control by warping the cell map. The field maps are converted to ramp modifiers, where the input values regionally scale the cell map. The cell map is then converted to a periodic gyroid lattice with ramped thickness using the same methods as the cell map.

Gyroid structures are characterised by their continuous, smooth surface that offers unique physical properties (Hanks et al. 2020). Gyroids exhibit good strength and deformation capability, allowing them to compress without catastrophic failure (Mechanical behaviour of tpms-based scaffolds 2019). These sheet-based structures, in comparison to strut-based structures, have smoother internal transitions, which lead to lower stress concentrations within the structure (Timercan et al. 2021; Wang et al. 2022). Additionally, a very large range of material properties is achievable by changing the unit cell geometric properties when compared to other types of lattice structures (Mechanical behaviour of tpms-based scaffolds 2019; Wang et al. 2022). Although the concepts outlined in this study would work for a variety of lattice structures, the gyroid was chosen as a case study and also for its suitability for the application.

**Fig. 12:**
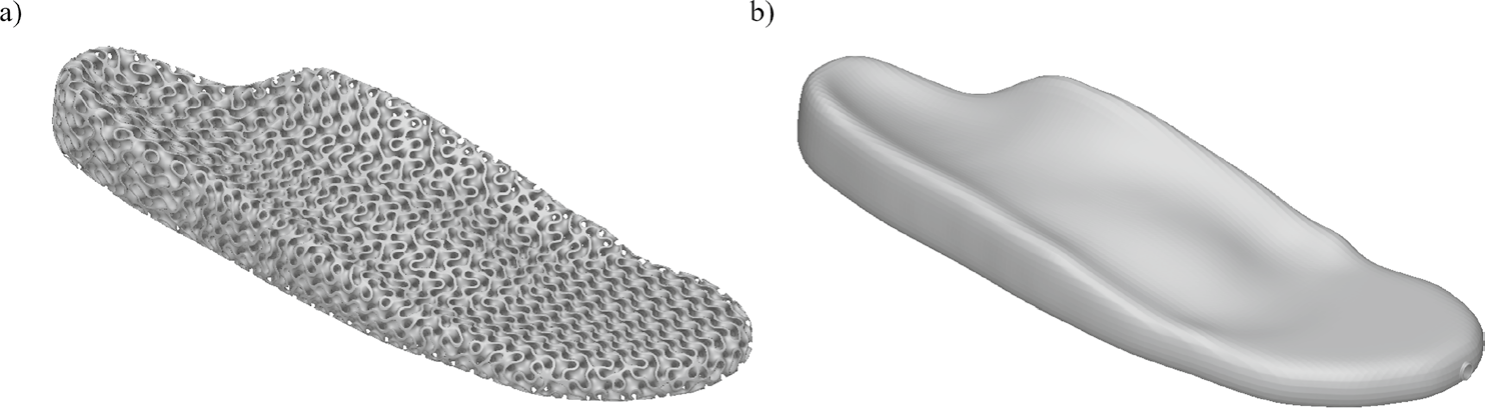
Final insole geometry **a** internal structure, and **b** full geometry

A boolean intersect is then performed with the cell map and the insole geometry to create the regionally tailored inner geometry. The inner structure of the insole is shown in Fig. 12a. A 0.5 mm thick shell of the insole was also created to incase the inner lattice. A boolean union was performed to combine the two parts. Next, two holes were created at either end of the insole to aid in post-processing the print (cleaning and filling). The holes had a radius of 2.5 mm, and matching plugs were also created to seal the holes separately. The final insole geometry is shown in Fig. 12b. Then the geometry was then meshed and exported as an STL.

**Fig. 13:**
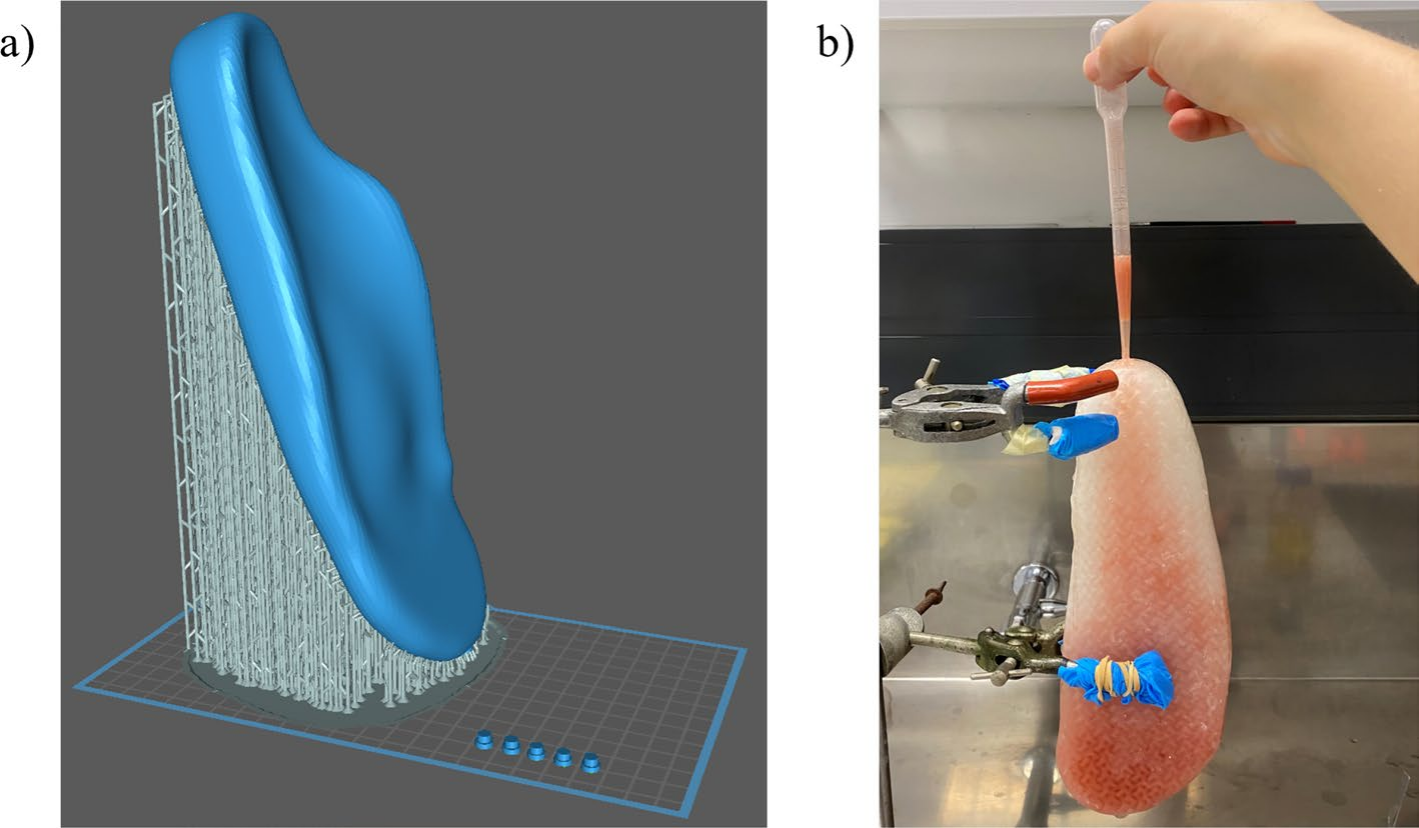
Manufacturing of the insole **a** print bed setup, and **b** filling the insole

The STL files were loaded into Chitubox version 1.9.5, a G-code preparation software. The insole was orientated at a 70-degree angle relative to the print bed. The insole could not be orientated to be at a 90-degree angle due to print height limitations. A difficulty associated with printing the insole was the printing consistency. Due to the weight of the insole, its position could change if the supports were not appropriately designed due to the highly flexible nature of the cured resin. A high density of manufacturing supports was implemented manually to ensure the position of the insole did not change and impact the quality of the print. Supports were designed to have connections with their neighbouring supports, minimising their likelihood of moving. The manufacturing supports and print bed layout are shown in Fig. 13.

The printer used was the Phrozen Mightly 8K SLA printer with Resione 39T resin. A four-second exposure time per layer was defined, aside from the initial layers, which had a 60-second exposure time to improve the bonding to the print bed. A 0.09 mm layer height was used. The print took 14 h to complete.

**Fig. 14:**
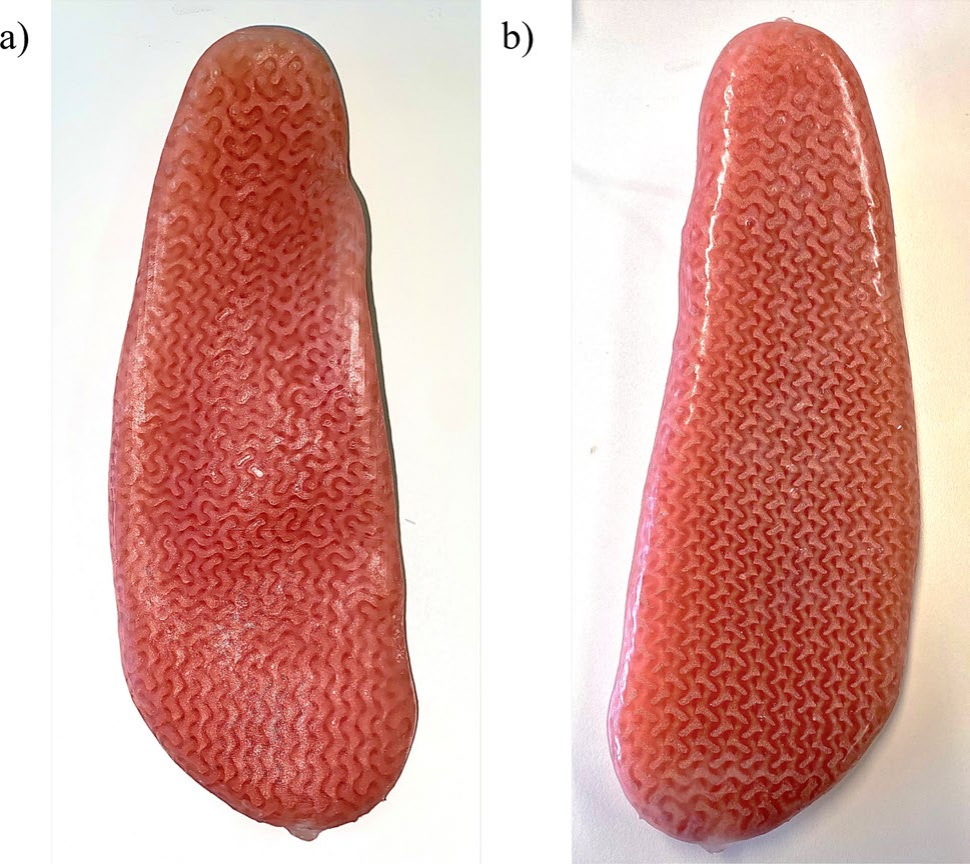
Final insole **a** plantar interface side and **b** ground facing side

After printing the insole, it was placed in a subsonic ethanol bath for five minutes. It was removed, and low-pressure compressed air was directed through one of the post-processing holes to remove any excess resin or ethanol. Subsequently, the hole at the toe end of the insole was sealed with one of the printed plugs. The plug was coated in a thin layer of uncured resin before being placed in the hole. Then, a UV curing pen light was used to cure the resin and seal that end of the insole. The insole was then secured in a retort stand with a single open hole at the top to fill it with silicone oil, as shown in Fig. 13b. Powdered pigment was mixed into the oil. This was an aesthetic choice that allowed the internal geometry to still be visible after manufacturing, as the silicone oil tended to obscure the internal structure. The final geometry of the insole is shown in Fig. 14.

#### Experimental analysis

Experimental testing of the insole was performed to compare its performance to the numerically modelled insole. The plantar pressure would be read while the subject walked. An F-Scan pressure map (model number 3010) was placed at the foot-insole interface to read the plantar pressure.

**Fig. 15:**
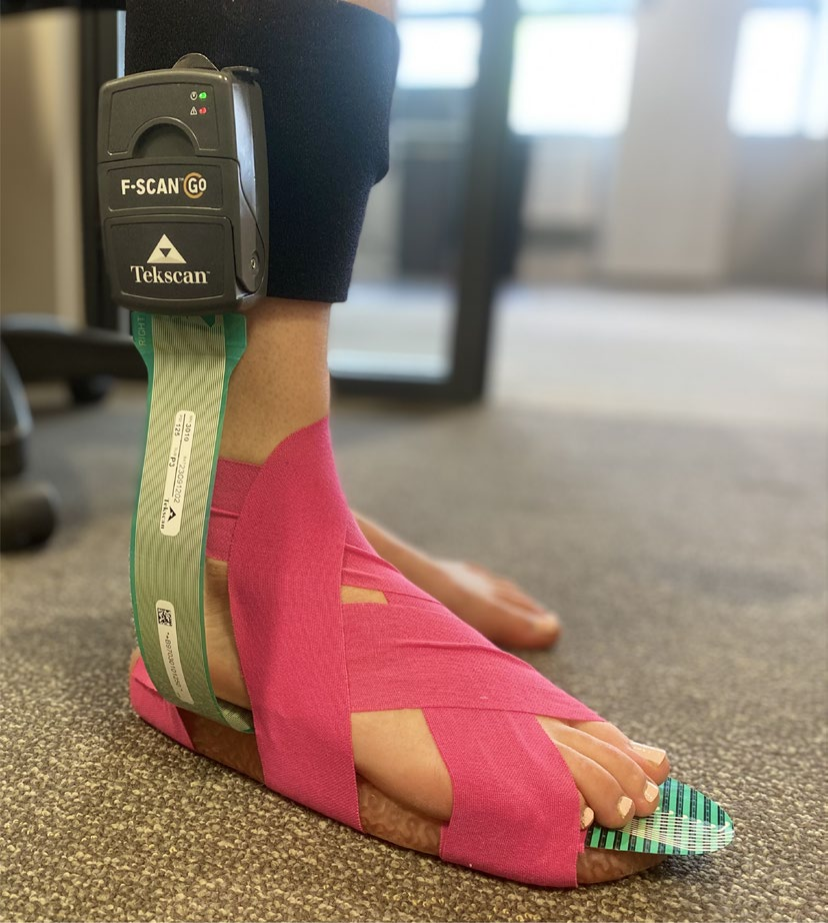
Insole testing setup

To minimise interference from additional factors, shoes were not used to fix the insole to the foot. Kinesiology tape from Sports Healthcare was used to assemble the foot, insole and sensor system. This tape would allow natural movement of the foot while firmly fixing the insole and sensor to the foot. The tape was adhered to the top surface of the foot while it was not stretched, allowing unrestricted movement. The tape was pulled into tension before being adhered to the insole. This tensioned application would restrict the movement of the insole relative to the foot, firmly securing it in place. The setup for this experiment is shown in Fig. 15.

## Results

### Gait simulating numerical model

#### Static model

**Fig. 16:**
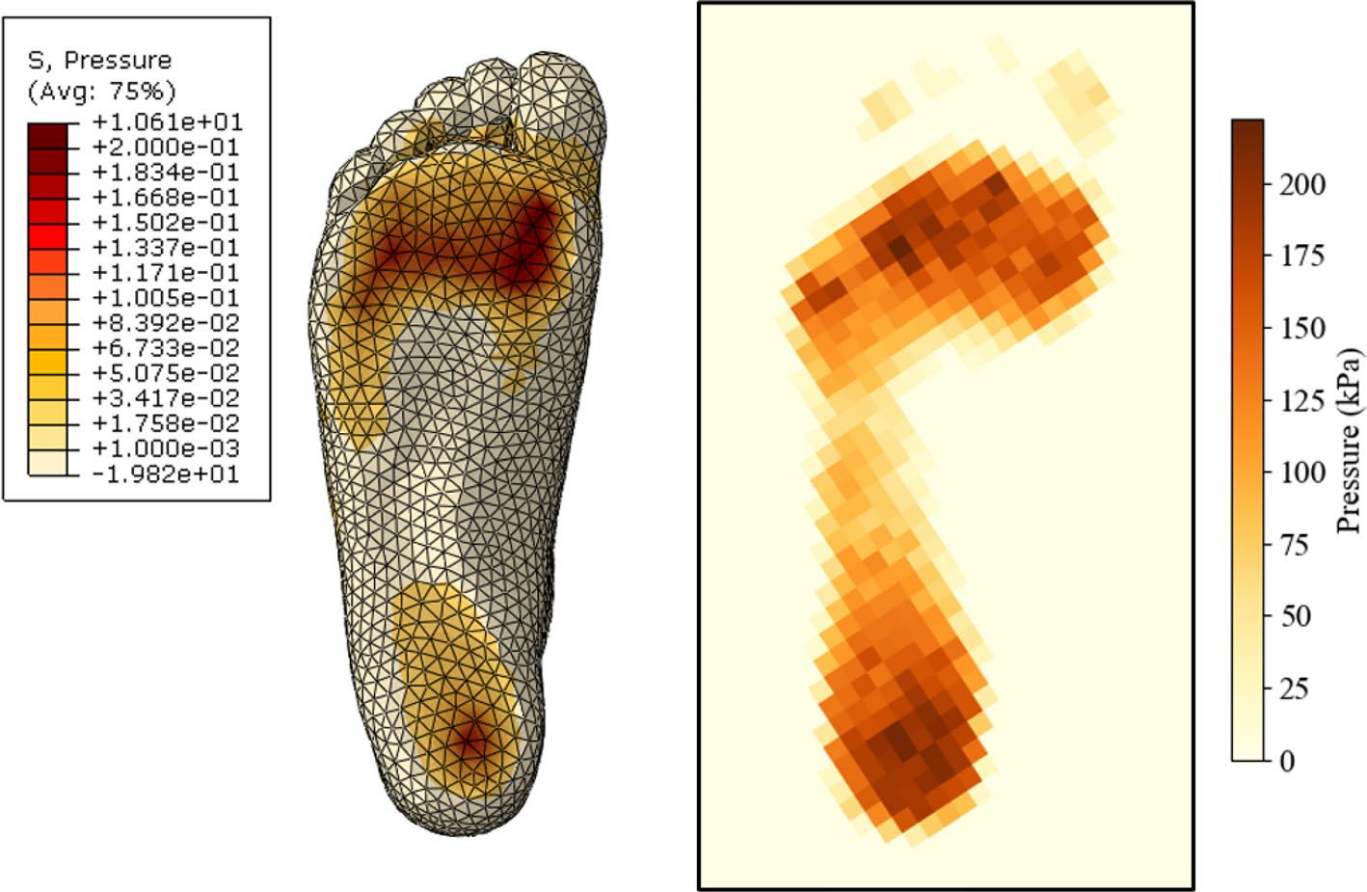
Static results for the numerical model and experimental analysis

The static model aimed to simulate the subject standing to verify the geometry and material properties provided an accurate representation. The results from the numerical model were compared to experimental pressure measurements, as shown in Fig. 16. The overall results show a similar pattern, where the peak plantar pressures are under the heel and the metatarsal heads. The magnitudes of the pressures are in the same range. The largest differences between the experimental and numerical results are the contact area and the stress concentrations.

In the numerical model, the peak pressure occurs under the first metatarsal head and tapers towards the lesser metatarsals, whereas the experimental results show the peak pressures under the heel and lesser metatarsals. The primary cause of this is the bone structure used in the numerical model. The subject’s bone structure used in Kathirgamanathan et al.’s study had significantly larger feet with longer phalanges (Kathirgamanathan et al. 2018). Scaling the bone structure to fit this studies subject’s foot led to inaccuracies with placement and position of the hard tissue structures.

The contact area in the numerical model is significantly lower, specifically in the midfoot region and the toes. This may have been caused by the casting methods and the nature of the simplified model itself. The foot was cast in an approximate standing position which captured the plantar topology accurately, however the toes were in a relaxed position. Further reduction in the contact area was caused by the approximation of the bone structure. With the forefoot bone system being fused into a lumped structure, the movement of the phalanges, and therefore the toes, was relatively stationary. The midfoot may have also been impacted by the simplification of the musculoskeletal structure, restricting the movement and flexion of the foot as a whole.

#### Dynamic gait model

#### Experimental plantar pressure measurements

**Fig. 17:**
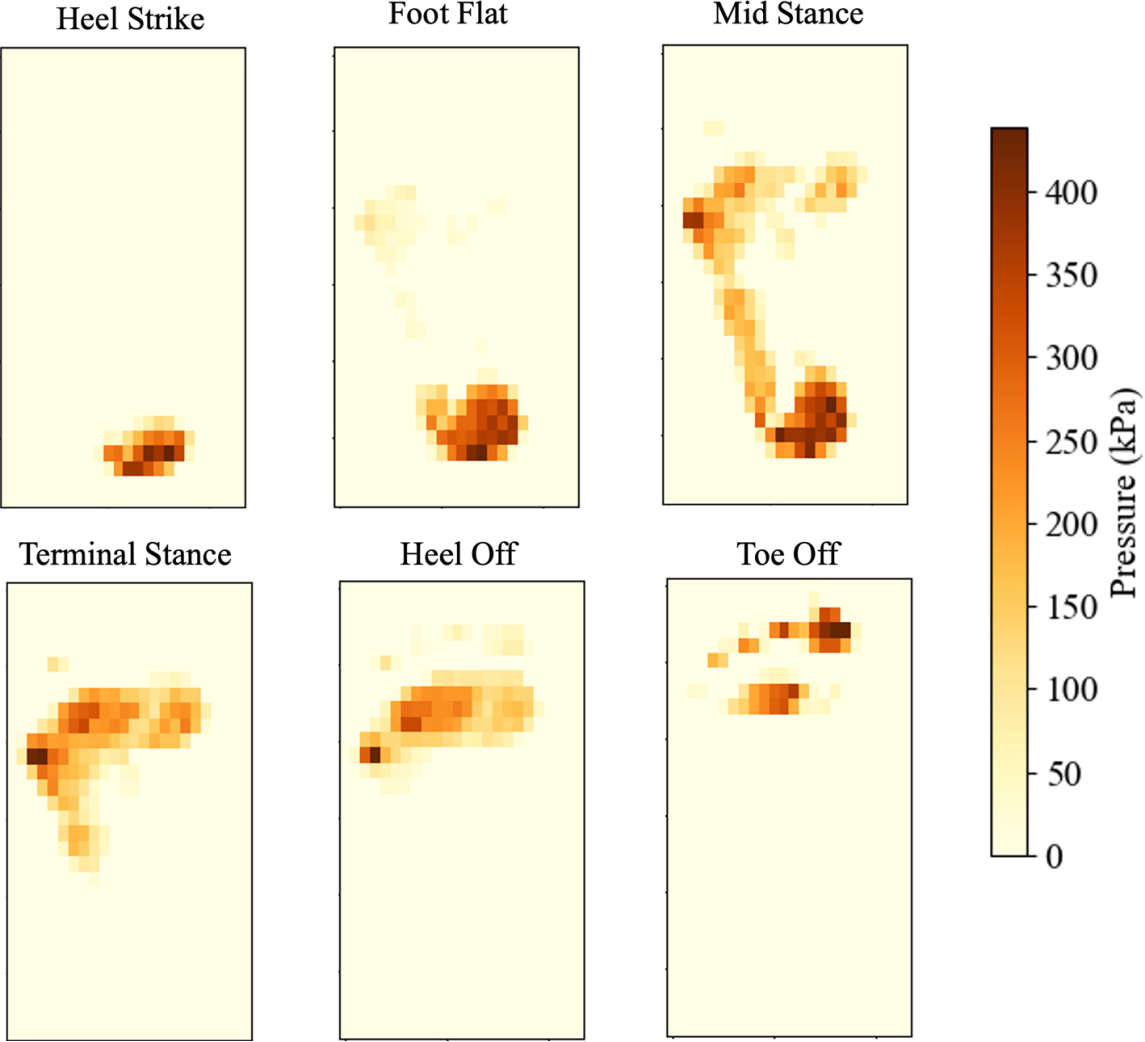
Experimental plantar pressure measurements taken at each stage of gait

Figure 17 shows the plantar pressure distributions for the key stages in gait. Overall, the results align with plantar patterns shown in literature (Cicciu et al. 2024; Chen et al. 2001), however the subject’s foot has a slight supination. The peak pressures observed in the dynamic pressure measurements are significantly higher than in the static model, where approximately double peak pressures are seen in every stage captured. This is caused by a smaller contact area carrying more load in most stages. It is well documented that from the end of the heel strike stage, the load rises and fluctuates between approximately half to the full load of the body weight until the end of the toe-off stage (Moreira et al. 2009; Qian et al. 2013; Ozmanevra et al. 2018; Alvarado-Rivera et al. 2022).

#### Numerical results

Table 2 shows the resulting reaction forces and moments at the proximal end of the ankle when the plantar pressure distributions are applied. The trend of the vertical and horizontal components of the reaction force show agreement with those published in literature (Qian et al. 2013), although it should be noted that the orientation of the coordinate system made it difficult to find a direct comparison. However, the general trend through each stage of gait gives confidence in these results.

**Table 2:** Resulting forces at the proximal surface of the ankle

Step	Heel strike	Foot slat	Mid-stance	Terminal stance	Heel off	Toe off
$F_x$ (*N*)	10.2	12.5	$-$ 4.0	34.3	$-$ 61.5	4.2
$F_y$ (*N*)	53.9	117.1	100.9	103.9	81.4	81.9
$F_z$ (*N*)	$-$ 116.1	$-$ 297.9	$-$ 290.7	$-$ 348.3	$-$ 513.6	$-$ 138.4
$M_x$ $(N \text {mm})$	$-$ 5867.9	$-$ 15466.2	$-$ 15070.4	$-$ 17908.6	$-$ 28331.7	$-$ 10579.2
$M_y$ $(N \text {mm})$	634.7	2164.2	2966.3	5079.4	7778.6	1167.9
$M_z$ $(N \text {mm})$	$-$ 188.9	220.8	1198.6	3279	4754.1	$-$ 1083.8

**Fig. 18:**
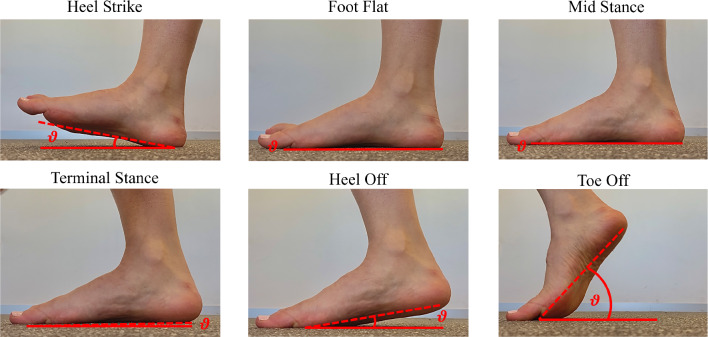
Frames of the video analysis used to calculate the contact angle

Key frames from the video analysis are shown in Fig. 18. The angle was taken by drawing an approximate line from the heel to under the metatarsals. The toes were not taken into account as their position moved significantly with respect to the rest of the plantar surface.

The resulting rotational speeds ($\omega$) are shown in Table 3. The speed at heel strike is kept at zero as the duration of this step in the numerical model initialises contact, so the rotation does not begin until the foot flat stage. To compensate for this, the initial angle that the ground is held at for the first step represents the angle at the completion of the heel strike phase.

From the end of the foot flat phase until the end of the terminal stance, there are minor contact angle changes. In these stages, the foot is closely aligned with the ground as the body weight shifts over it, as shown by the foot-to-ankle angle in Fig. 18. The most significant rotation occurs in the toe off stage where the most contact occurs in the toes, where their pliability allows for a greater contact angle. These of these results align with the general pattern found in literature (Qian et al. 2013; Moreira et al. 2009; Cicciu et al. 2024). It should be noted that the more common method to simulate ground rotation is by using two separate axes, one around the hindfoot and one about the forefoot.

**Table 3:** End point contact angle for each stage of gait

Step	Heel strike	Foot flat	Mid-stance	Terminal stance	Heel off	Toe off
$\omega$ (radians)	0	$-$ 0.815	$-$ 0.123	$-$ 0.175	$-$ 0.62	$-$ 1.87

**Fig. 19:**
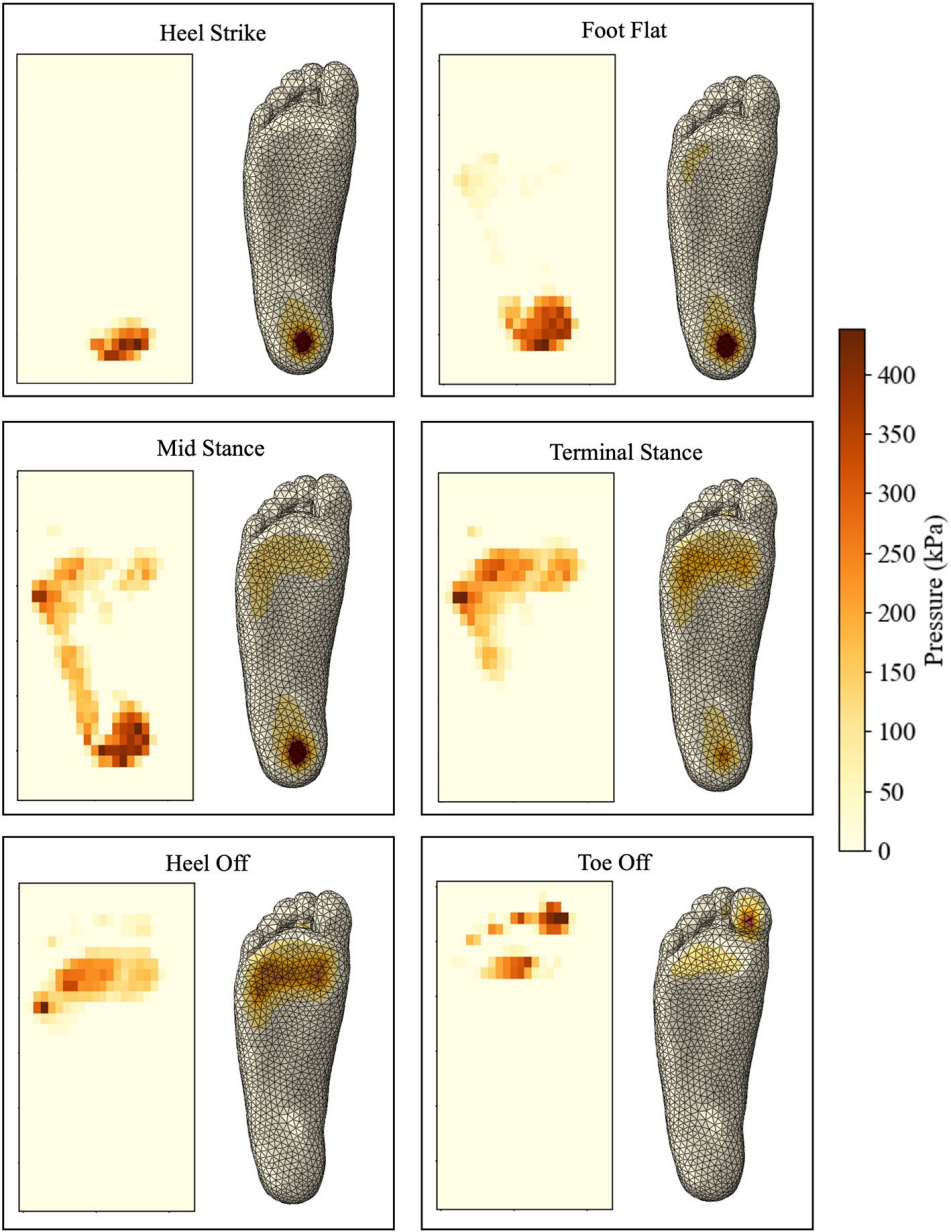
Numerical and experimental plantar pressures for each stage of gait

Figure 19 shows the numerical pressure distribution compared to the experimental results. Overall, the location of the peak pressures is replicated well in the numerical model in each stage. The primary difference across all stages is the concentration of pressure.

**Fig. 20:**
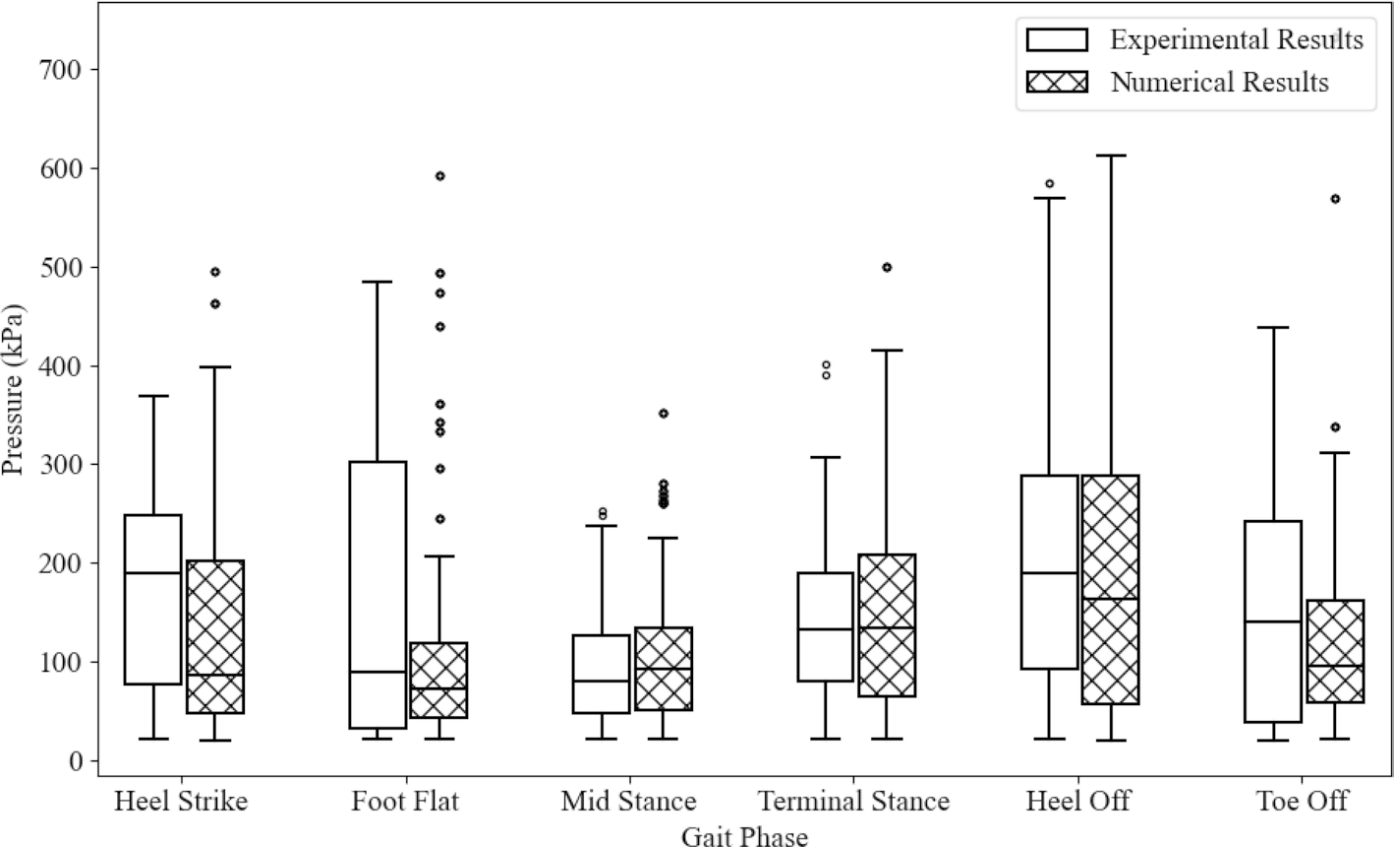
Distribution of plantar pressure for experimental and numerical results

Figure 20 shows the distribution of pressure values for the numerical and experimental results. The overall agreement varies across different gait phases. There is a general trend of the numerical results underestimating the pressures, particularly in the initial heel strike and foot flat stages. The median pressures become closer in the mid-stance and terminal stance. The presence of outliers in numerical results, particularly in the foot flat stage, suggests high-pressure regions in numerical simulations. This may be due to the geometry and material definitions in the model not allowing the foot to conform more realistically to the ground. Conversely, it could indicate that the sensor had been previously damaged, causing certain sensors to measure pressures incorrectly. Numerical results generally exhibit greater variability, whereas experimental results tend to have lower median pressures and fewer outliers. The phases with better agreement (mid-stance and Terminal Stance) could indicate that the numerical model more accurately captures the mechanics of these particular gait phases.

Table 4 shows the difference between experimental and numerical results in terms of the mean difference, quartile one difference and quartile three difference. There is a significant difference in the first two phases; the mean difference in heel strike and the quartile three difference in foot flat. Both of these large differences are a higher measurement in the experimental data. This may be from the concentrated stress in the numerical model where the foot appeared to not conform to the ground as much. Overall, the other differences are minor in comparison and it is suspected that the higher difference may be due to the same difference in the numerical model.

**Table 4:** Experimental and numerical differences between the median, quartile one quartile three for each stage of gait

	Heel strike	Foot flat	Mid-stance	Terminal stance	Heel off	Toe off
Median Difference (kPa)	104.79	17.41	13.47	1.99	27.26	44.29
Q1 Difference (kPa)	29.67	10.98	3.39	15.21	35.76	19.24
Q3 Difference (kPa)	46.48	183.07	8.47	18.31	0.4	80.44

### Optimisation algorithm for insole design

#### Static optimisation

**Fig. 21:**
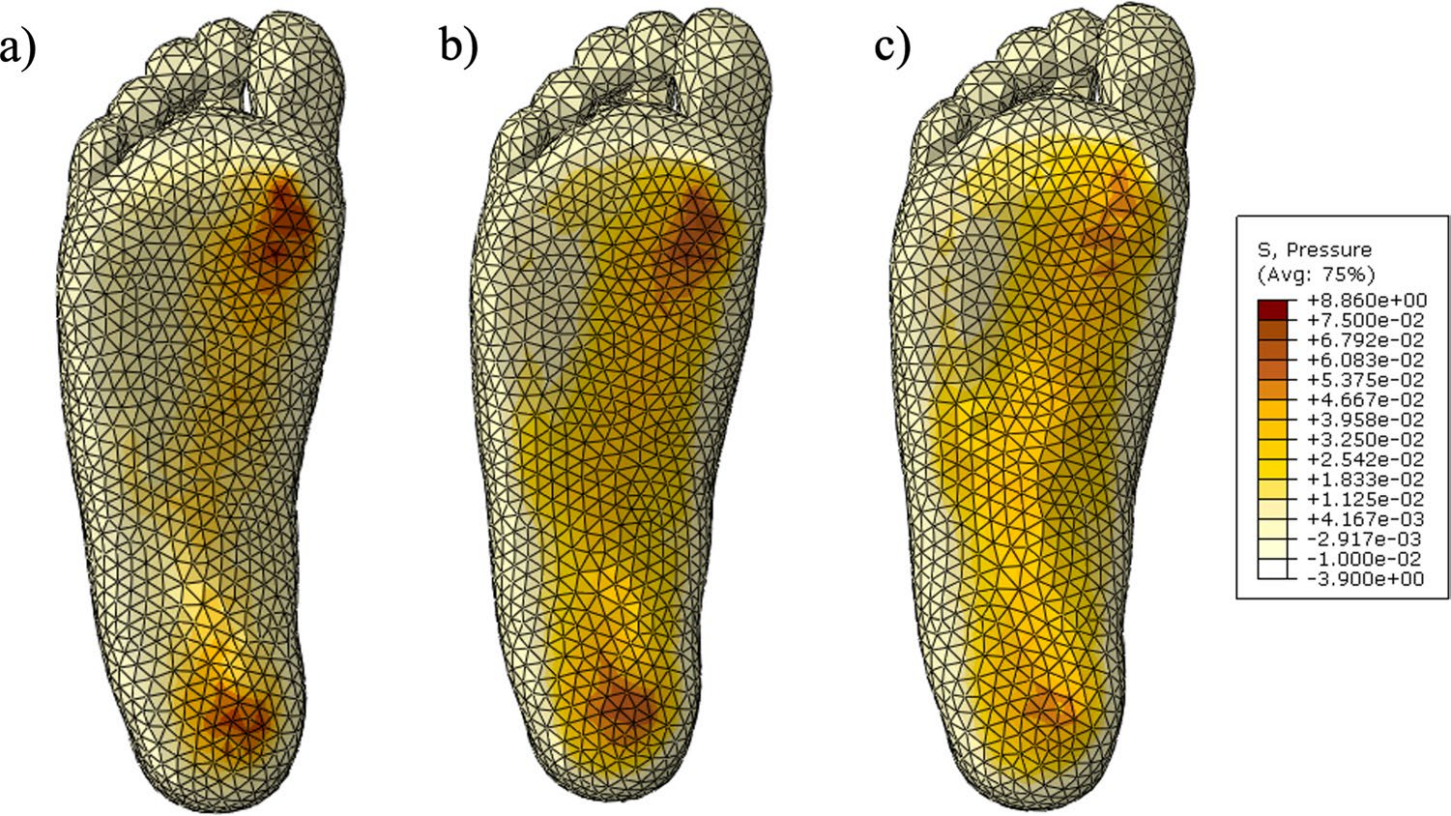
Static analysis results. **a** Initial lattice with no fluid, **b** starting parameters in optimisation algorithm, and **c** statically optimised insole

One hundred twenty-seven jobs were submitted in the static optimisation process. The total process took 17 h and 40 min. The process ended when no significant change in the objective function was found by further changing the regional geometry. Figure 21 shows the plantar pressure of the static model with an insole at various stages.

Figure 21a shows the results for the initialised insole geometry without fluid added. There are two major stress concentrations at the first metatarsal head and under the heel. There is a secondary high-pressure band running from the first metatarsal to the heel. This may indicate that too much load is being carried by the arch for this insole geometry.

Figure 21b shows the insole at the initialised stage of the optimisation process for a fluid-filled insole. A significant improvement can be seen in comparison to the insole without fluid, with more dispersed pressure and a lower magnitude in the peaks. Slightly more area is carrying load, particularly in the forefoot region. This may be due to the SLC insole having the ability to change its shape more freely. The fluid may be able to flow away from the high-stress area, particularly the arch, and replace the fluid volume in other lower-pressure regions.

Figure 21c shows the optimised insole. There is a significant reduction in the peak pressure regions (first metatarsal and heel) in size and magnitude. The lesser metatarsals appear to take more load, which may be offloading those high-pressure regions.

**Fig. 22:**
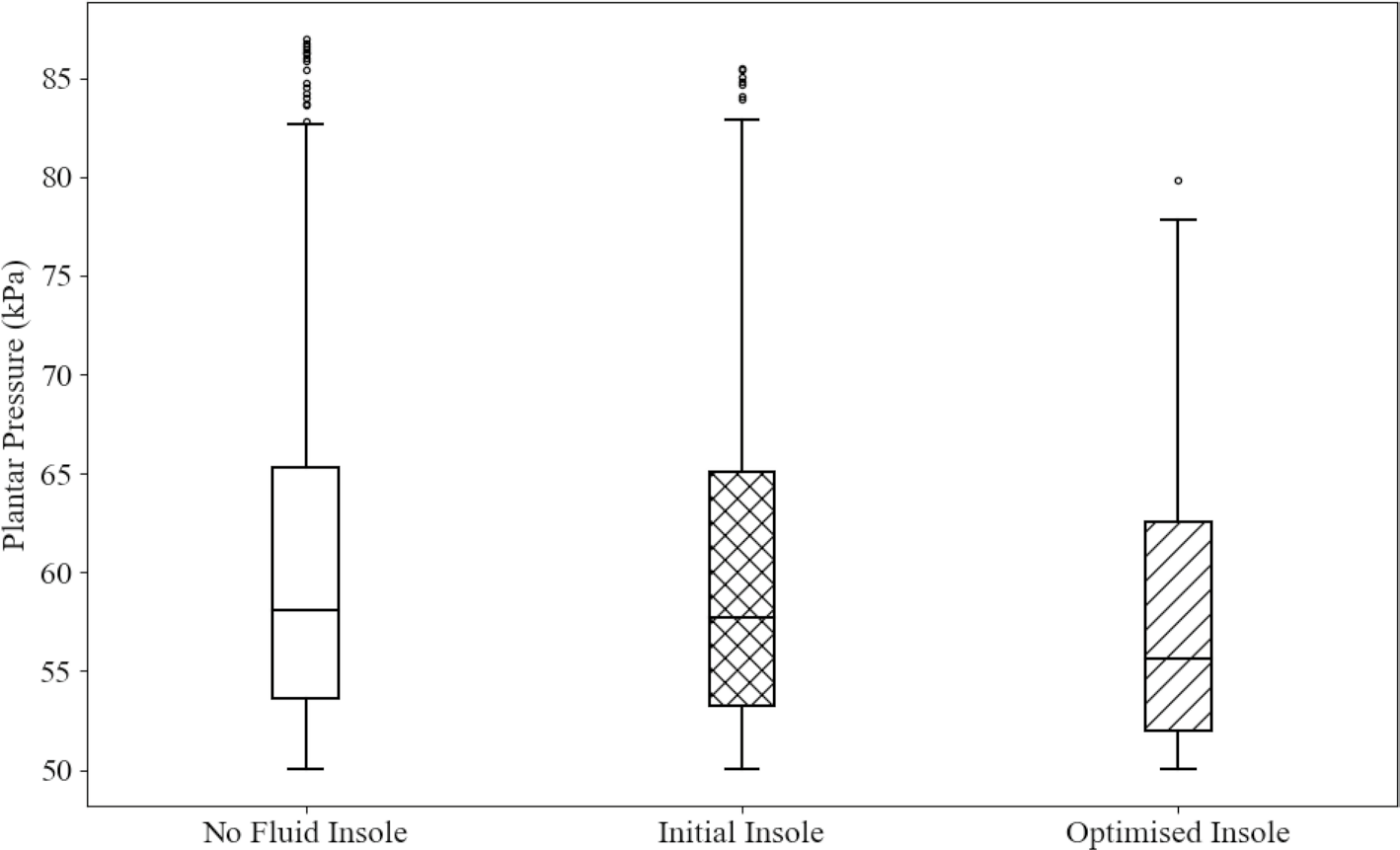
Plantar pressures for the different static models

Figure 22 compares plantar pressures between a fluidless insole and a fluid-filled insole with the initialised geometry and the optimised fluid-filled insole. One of the most notable comparisons is between the initial fluidless and fluid-filled insoles. The addition of the fluid does not appear to make any significant differences in the overall pressure distribution. This may be because the insole geometry was already in total contact, meaning the fluid did not impact the contact area. However, the fluidless insole has more outliers on the upper end, reaching a higher maximum stress of approximately 87 kPa in comparison to the fluid-filled insoles of 85.5 kPa.

**Fig. 23:**
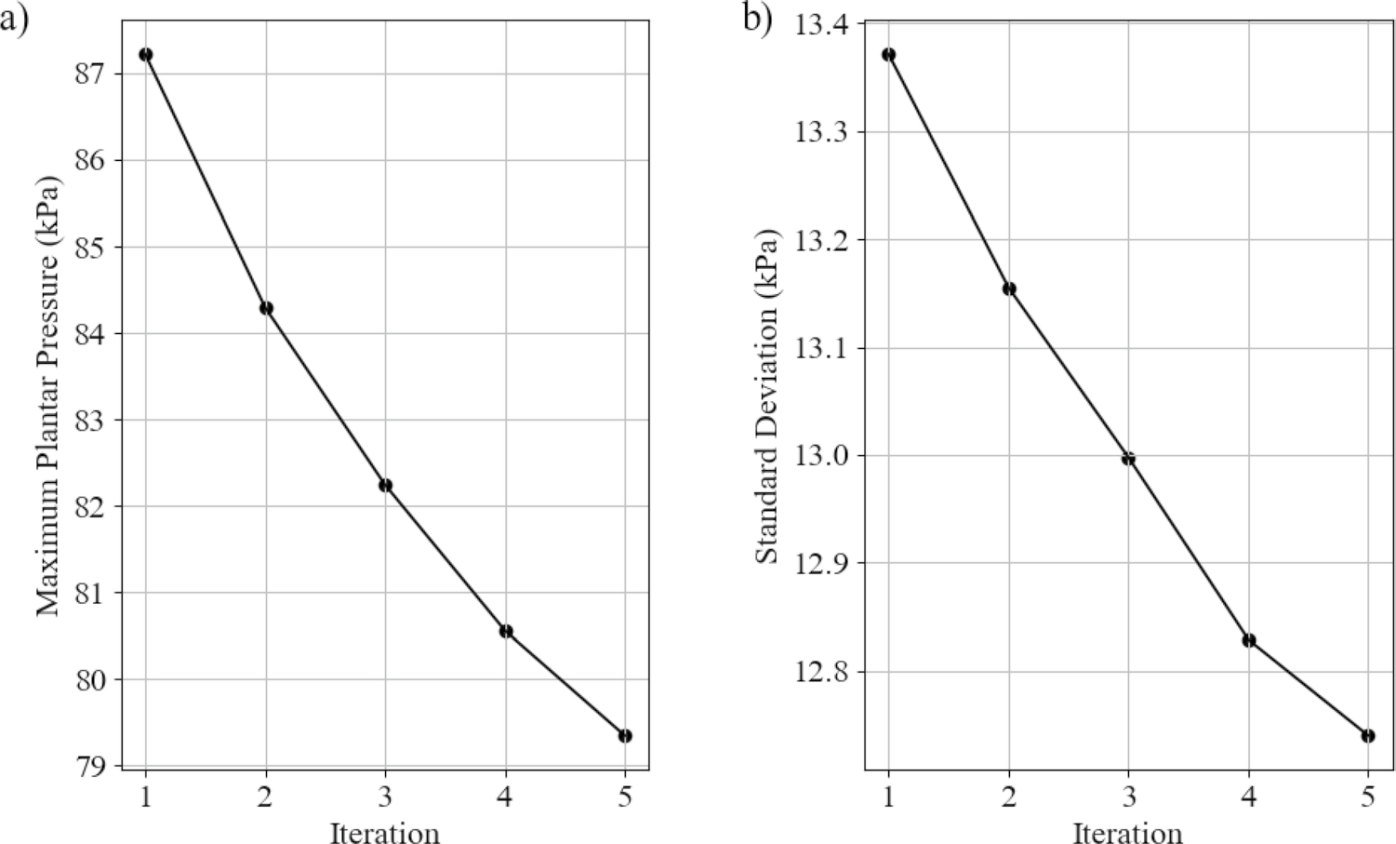
Optimisation results for each iteration. **a** objective function value with iteration and **b** standard deviation of plantar pressure

Figure 23 shows the change in the objective function value (maximum plantar pressure) and standard deviation of plantar pressure. The maximum planar pressure had a reduction of approximately 8%, whereas the standard deviation of pressure had a reduction of approximately 5%.

**Fig. 24:**
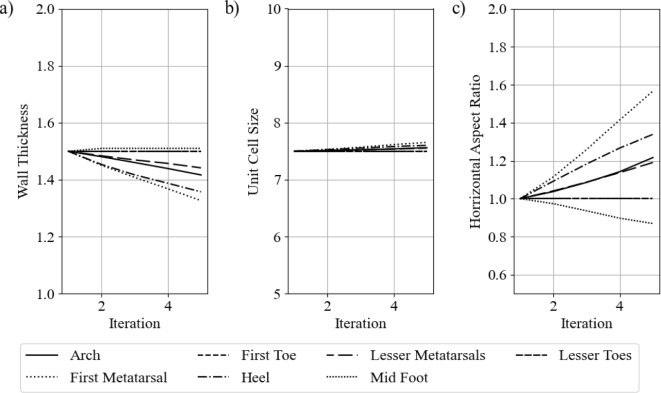
Geometric parameter changes with iterations. **a** Cell wall thickness, **b** unit cell size, and **c** horizontal aspect ratio

Figure 24 shows the change in geometric parameters with iterations. The axis limits have been set to the upper and lower bounds to show the extent of the changes that occurred.

The first notable trend is that both of the toe regions (first toe and lesser toes) did not have any change from their initial values. Results showed that due to the design of the insole geometry, the toes never came into contact with the insole itself, meaning they would have no influence on the maximum stress.

The unit cell size did not have a significant change in any of the regions in comparison to the wall thickness and horizontal aspect ratio. This may be because more significant material property changes can be achieved with the latter variables. As discussed in previous work, between the defined geometric bounds a percentage change in the wall thickness has a greater impact than the same percentage of change in the cell size (Cracknell 2024). The general principles are that the stiffness of a structure can be decreased by decreasing the wall thickness, increasing unit cell size, or increasing the horizontal aspect ratio. The inverse applies to making a stiffer structure.

Figure 24 shows that all regions changed to become softer, aside from the midfoot region. This suggests that the midfoot needed to offer more support to offload the other regions. The most significant changes occurred in the first metatarsal region and the heel region. These results align with the initial peak stiffness seen in Fig. 21.

#### Dynamic optimisation

**Fig. 25:**
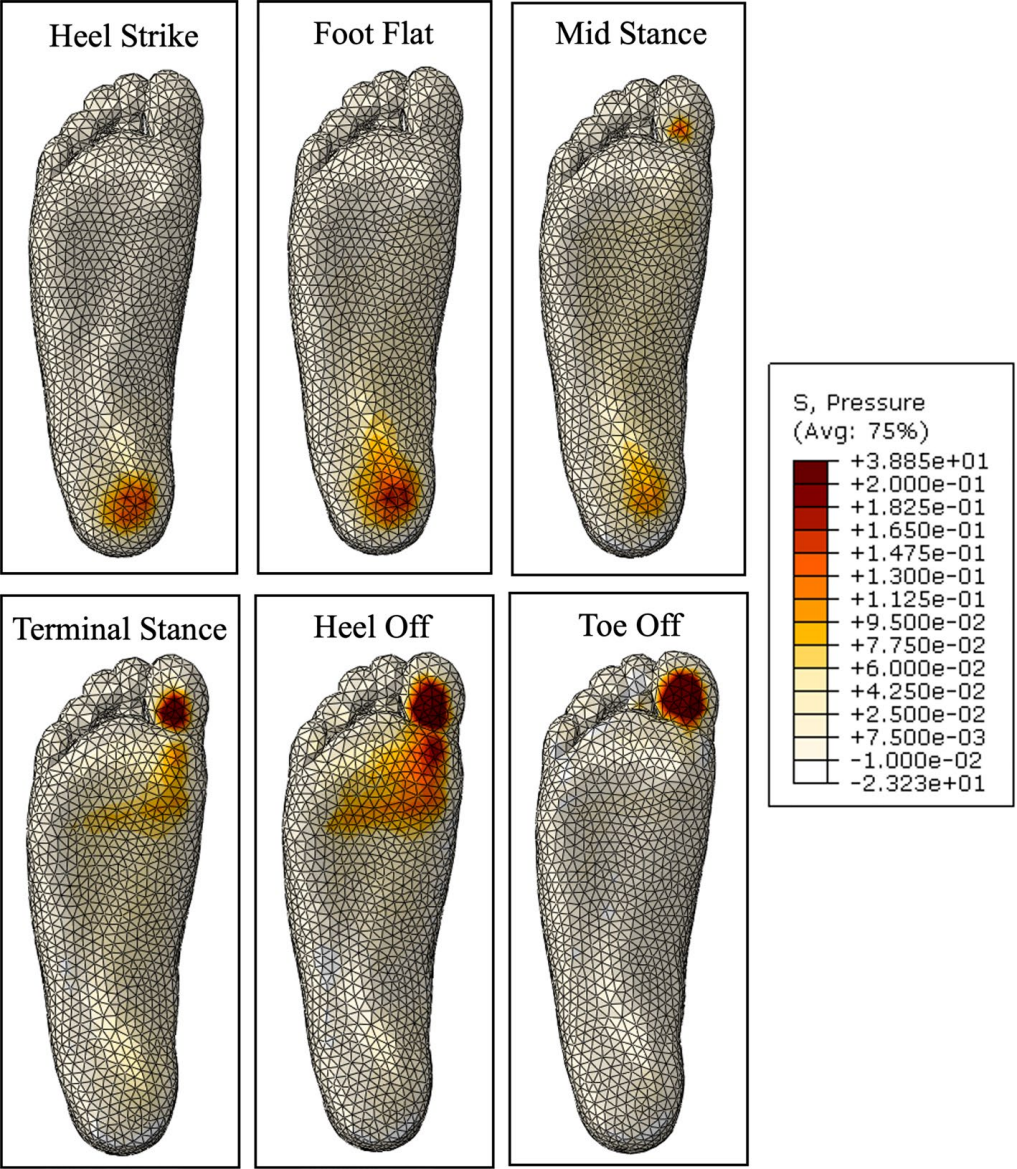
Plantar pressure through gait with initial insole geometry with no fluid

Before the optimisation process, a fluid-filled insole with the initialised geometry was compared with an identical insole with no fluid. Figure 25 shows the plantar pressure distribution through gait, wearing an insole with no fluid. The addition of a total contact insole shows a significant improvement from the peak pressures that occur without an insole, particularly in the foot flat to terminal stance stages. There is more support in the arch, which offloads the heel and metatarsal heads. In the heel off and toe off stages, there is still a large peak pressure on the first toe.

**Fig. 26:**
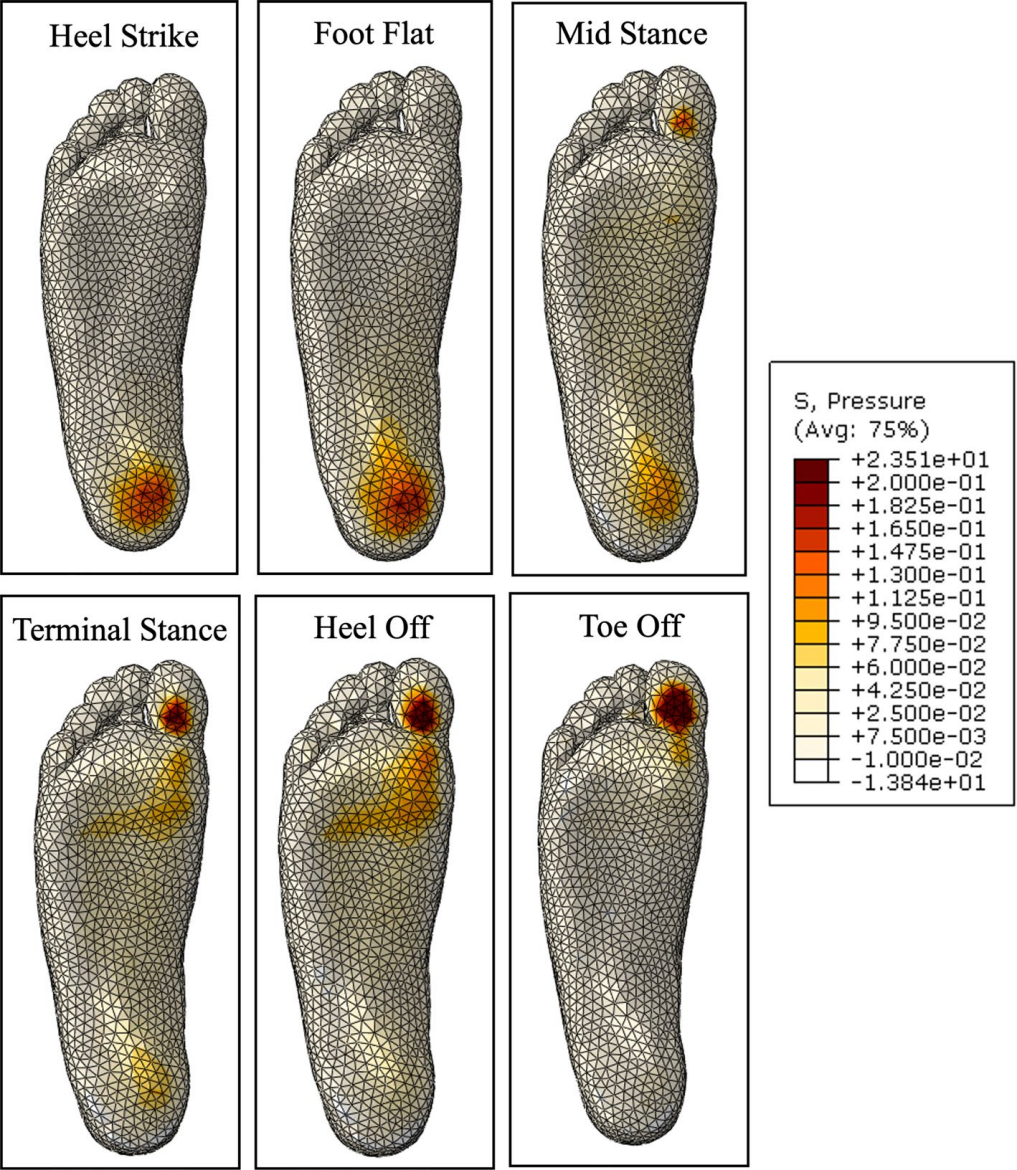
Plantar pressure through gait with initial insole geometry with fluid

Figure 26 shows the plantar pressure distribution through gait with the initialised fluid-filled geometry. The distribution is similar to the fluidless insole results, particularly from heel strike to terminal stance. There is a small increase in high pressure areas, resulting in lower peak magnitudes. The largest difference occurs in the heel off and toe off stages. The heel off stage shows more area still in contact with the insole, and a significant reduction in pressure magnitudes at the metatarsal heads. This suggests that the fluid was driven out of this area and inflated other areas, offloading the peak pressures. A similar result can be seen in the toe off stage, where the metatarsal heads are still carrying a small load reducing the peak pressure area.

**Fig. 27:**
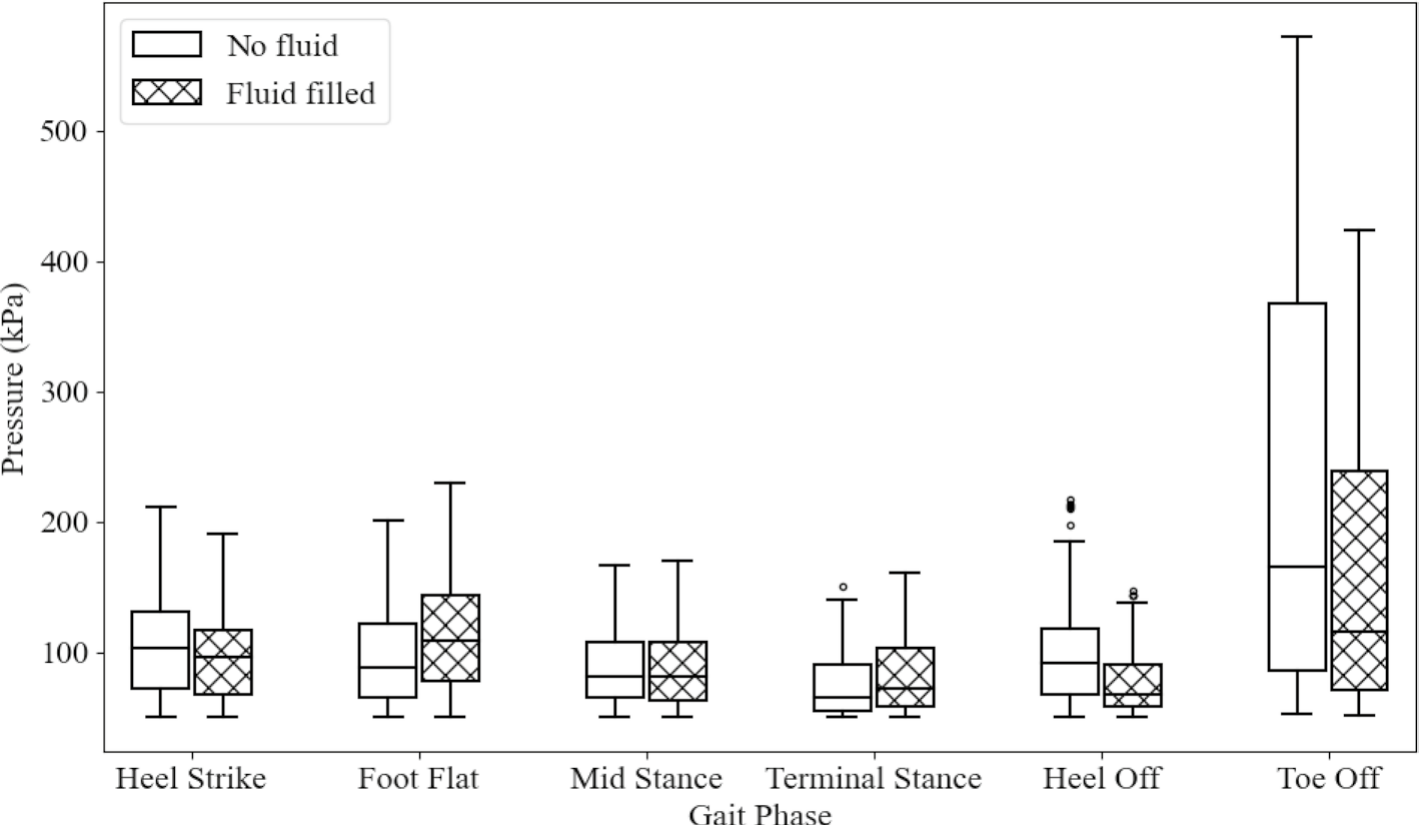
Comparison between initial insole geometry with fluid and with no fluid added

Figure 27 compares plantar pressures throughout gait for the initial insole geometry, comparing a fluidless and fluid-filled insole. The first four stages have similar results with only minor differences. The largest changes are in the heel off and toe off stages. The difference in the heel off median is 23.6 kPa, and a third quartile difference of 27.8 kPa. The toe off stage has a median and third quartile difference of 49.1 kPa and 129.1 kPa, respectively.

These results show that fluid-filled insoles do not make much difference in lower peak pressure gait stances but significantly reduce the peak and mean pressures in high peak pressure stances, particularly heel off and toe off.

**Fig. 28:**
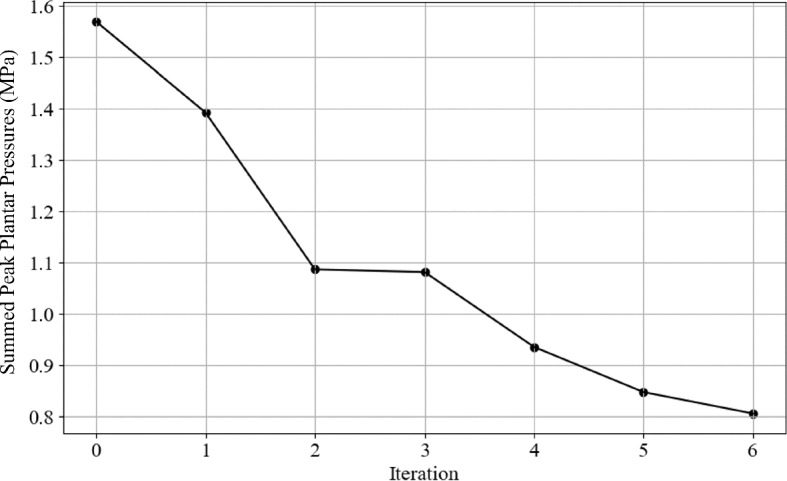
Summed plantar pressure through gait with optimisation iterations

The optimisation process took 57 h and 37 min and submitted 177 jobs. The dynamic model took significantly longer to run than the static model, explaining the time difference. Figure 28 shows the change in objective function value with increasing iterations. The dynamic model found its optimal value in six iterations, with an approximate 50% reduction in peak pressures.

The change in summed peak plantar pressures between the second and third iterations is significantly smaller than all other iterations. At the third iteration, the algorithm initially stopped as there was no significant change in the objective function value. The output files showed that this was not the only change in some variables that showed further significant reductions, but the applied direction vector resulted in a minimal change in the summed pressures. At this point, the algorithm was modified to create a direction vector that changes only the most influential parameters rather than every parameter changed by differing amounts. After this modification, further reductions in the objective function value were achieved until iteration six, where no significant change was found by further modifying the parameters.

**Fig. 29:**
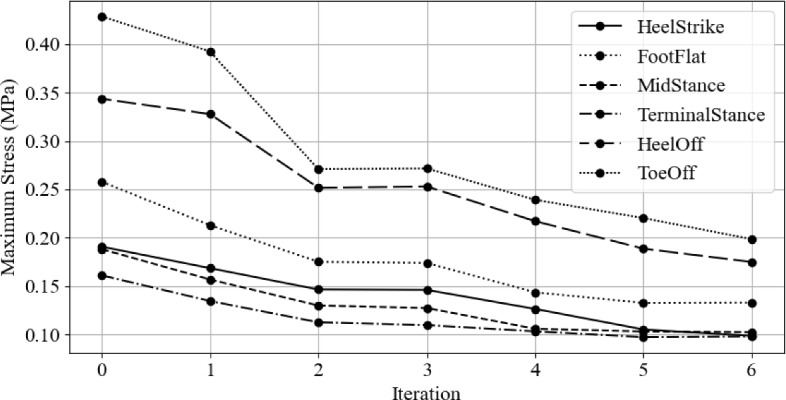
Maximum plantar pressure for each gait stage with optimisation iterations

Figure 29 shows the gait stage-specific changes in peak plantar pressures with optimisation iterations. The heel off and toe off stages have the largest peak plantar pressures throughout the optimisation process, and they also have the most significant reduction.

Mid stance and terminal stance had little change after the second iteration. The initial peak stress in these stages was small relative to the other peak pressures. This may be because the foot is approximately in total contact with the insole, resulting in a good pressure distribution and, therefore, peak pressures.

**Fig. 30:**
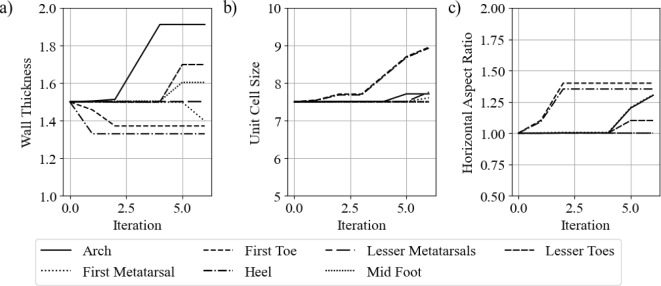
Lattice geometry parameter changes, **a** wall thickness, **b** unit cell size, and **c** horizontal aspect ratio

Figure 30 shows the parameter change in each region with each iteration of the optimisation process.

The arch region had very little change in cell size or horizontal aspect ratio, as shown in Fig.  30b, c. However, it has a large change in the wall thickness, as shown in 30a. This results in a much stiffer arch region, providing more support and offloading the high-pressure regions.

The midfoot region had very little change in all parameters up until the fifth iteration. This suggests that it had minimal influence on the maximum plantar pressures through gait up until other regions, such as the first metatarsal and toe, became relatively soft. The midfoot becoming slightly stiffer created more of an offloading effect.

The heel and first toe also have significant changes. Both have a large reduction in wall thickness in the first two iterations, coupled with an increase in the horizontal aspect ratio. Both of these changes result in a softer material. At iteration three, there is a sudden increase in unit cell size in both regions. This also makes the region softer.

The first metatarsal region had very little change before iteration five. At the final iteration, it had a decrease in wall thickness and an increase in unit cell size and horizontal aspect ratio. All of these parameter changes lower the stiffness of the structure.

The lesser toes and lesser metatarsals had very minimal changes throughout the optimisation. They have very little contact with the insole, meaning they would have little influence on the objective function.

**Fig. 31:**
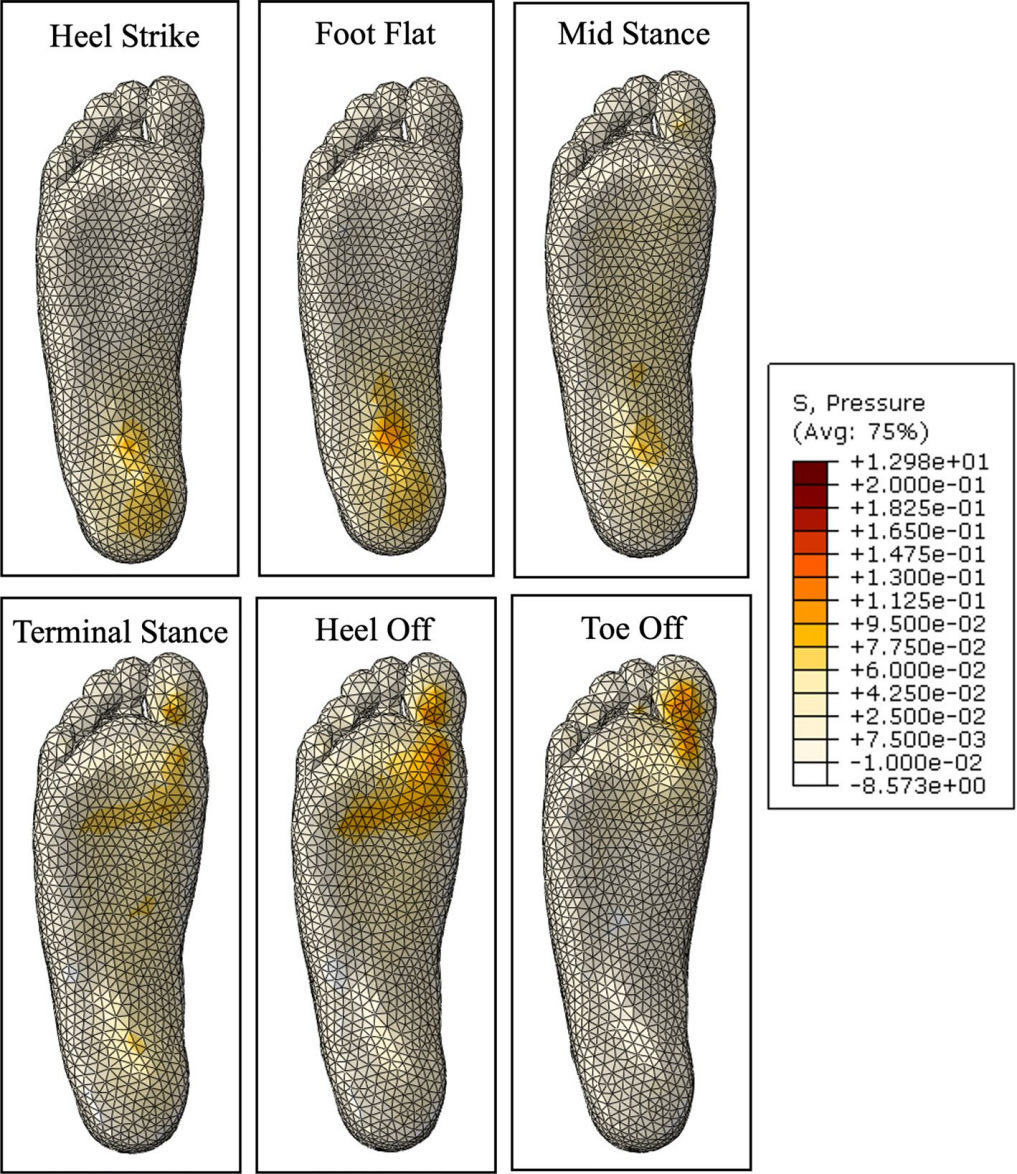
Plantar pressure through gait with optimised insole geometry

The plantar pressures through each stage of gait with the optimised insole are shown in Fig. 31. The magnitude of peak pressures through each stage of gait is visibly clear, particularly in heel strike, foot flat, heel off, and toe off. This may be due to the significant reduction in insole stiffness in the high-pressure regions. There is also a slightly higher area in contact, allowing the load to be more evenly distributed. This may be because the high-stress regions generally became softer, requiring less fluid pressure to inflate.

Another change can be seen in the region where the arch and heel connect. There is a stress concentration that is not seen in the initial geometry results. This is because the arch region became much stiffer, so the peak pressure is carried by the heel end of the arch.

**Fig. 32:**
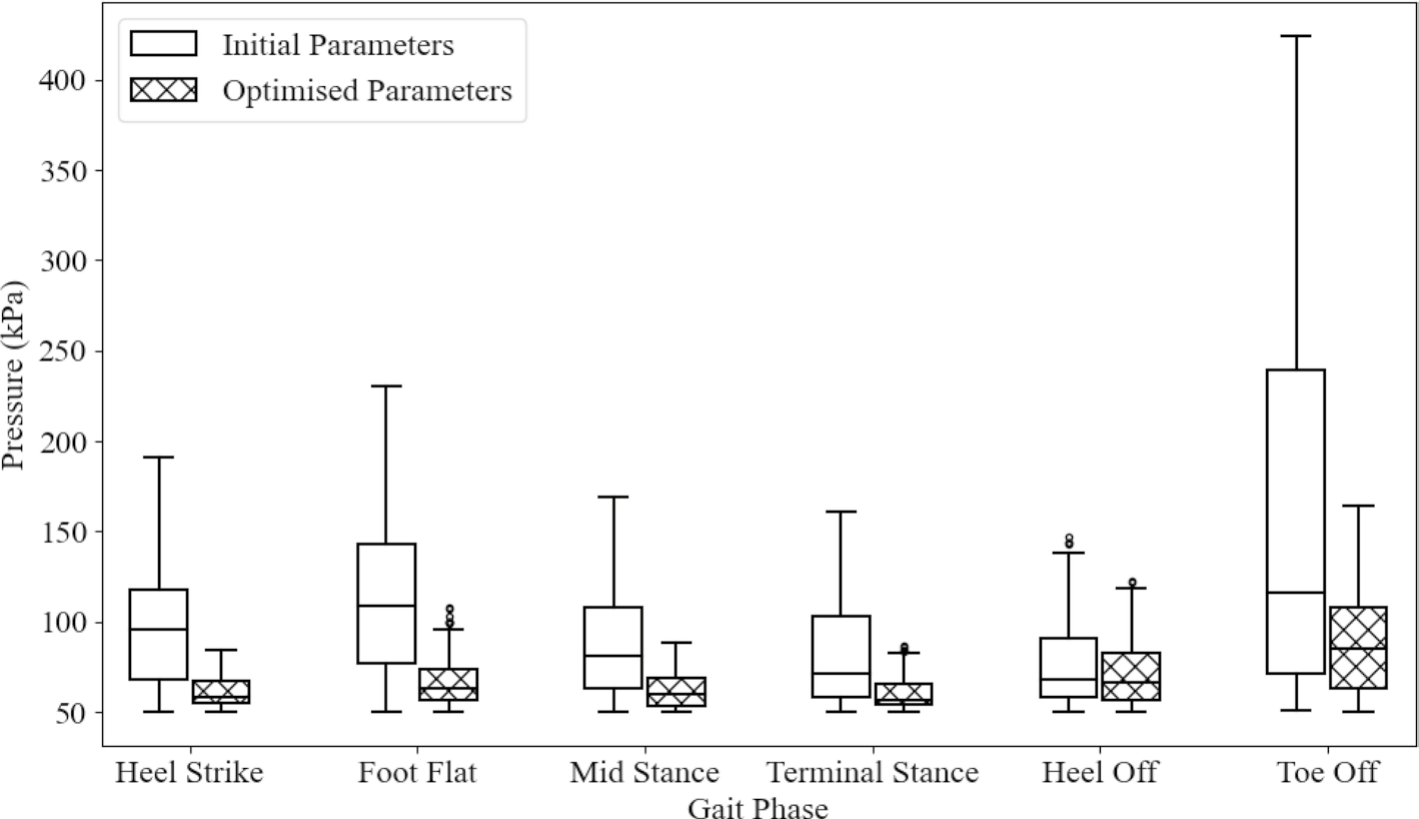
Plantar pressures through gait for the initial and optimised insole

The differences in plantar pressure are shown in Fig. 32, and the quantified differences in the median, first and third quartile are shown in Table 5. The most significant relative reductions can be seen in the heel strike, foot flat and mid stance phases. The smallest change can be seen in the heel off stage, where peak pressures were reduced, but the average pressure remained approximately the same. This may be due to the reduced offloading effects from the arch. However, the toe off stage had a significant reduction in peak pressure and a slight reduction in the average. This suggests that the peak toe pressure was able to be reduced by modifying the material properties, however, the load taken by the metatarsals in the heel off stage cannot be significantly changed.

**Table 5:** Differences between the initial insole and the optimised insole in their median, first quartile and third quartile for each stage of gait

	Heel strike	Foot flat	Mid stance	Terminal stance	Heel off	Toe off
Median difference (kPa)	37.5	45.7	21.2	14.65	1.6	31.1
Q1 Difference (kPa)	13.1	20.5	10.0	4.5	1.7	8.2
Q3 Difference (kPa)	50.1	69.7	39.2	37.2	8.3	130.9

### Insole performance

**Fig. 33:**
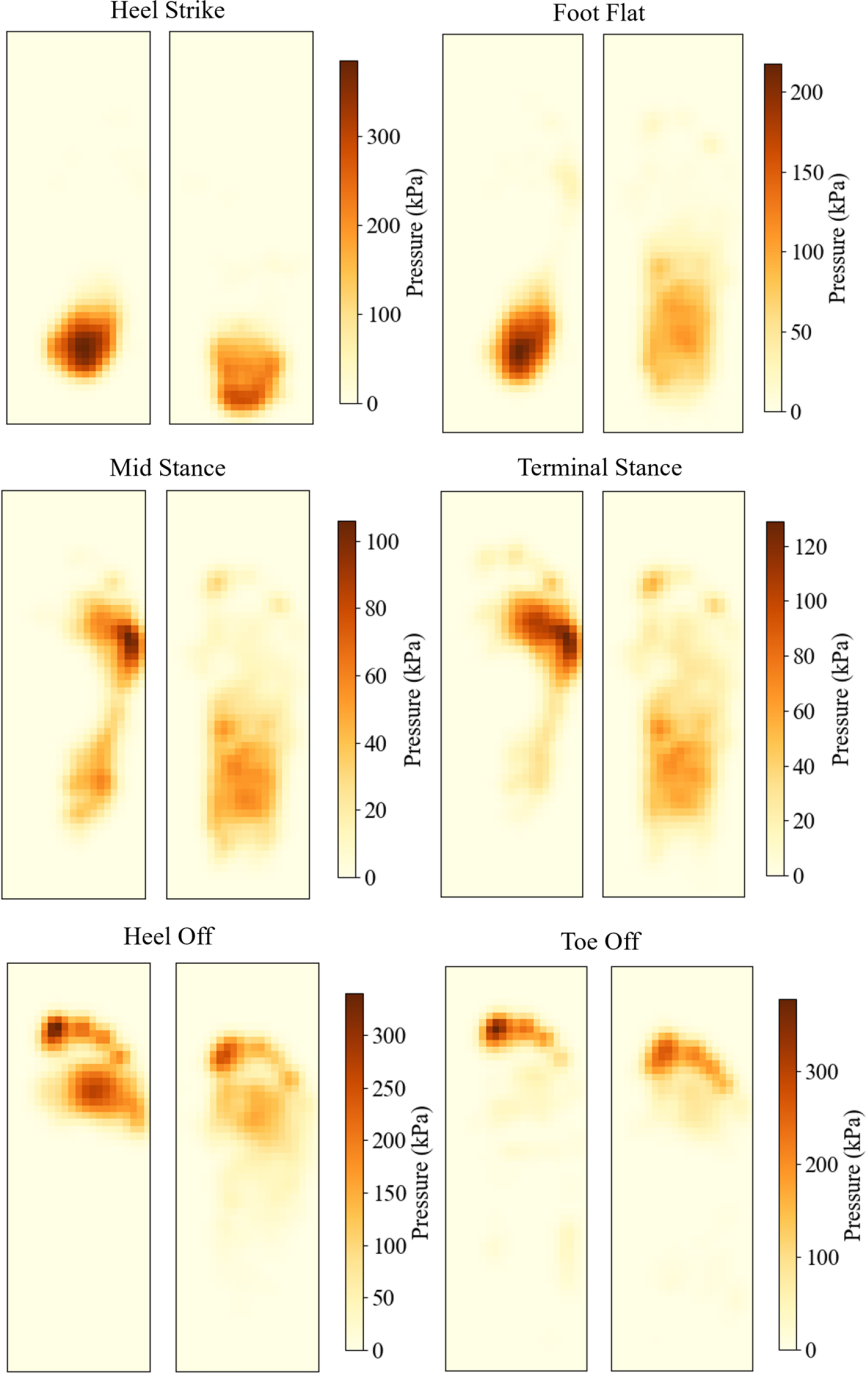
Experimental plantar pressures through gait. Barefoot pressures on the left, and insole plantar pressures on the right

The pressure maps from the experimental analysis are shown in Fig. 33. The raw data was processed by applying a Gaussian filter, as small sensor errors made the results less clear. In general, it is evident that across every stage of gait, the insole pressures have much lower magnitudes and are more evenly spread.

The heel strike pressure maps do not have a significant difference in contact area with the sensor, but the high peak pressure under the heel is reduced.

During the foot flat stage, the insole pressure map shows a significant portion of pressure being carried by the arch, whereas the barefoot counterpart still has a large peak pressure under the heel.

The midstance and terminal stance show that the barefoot peak pressure moved to the lesser metatarsal, while the majority of the pressure with the insole is still being carried by the arch. There is very little pressure in the metatarsals as the arch offloads them.

The heel off and toe off stages have a large peak pressure under the first toe in the barefoot results. The peak pressure under the metatarsals moves towards the middle during the heel off stage. The insole pressure maps show significantly lower peak pressures. However, it is similarly located under the first toe.

**Fig. 34:**
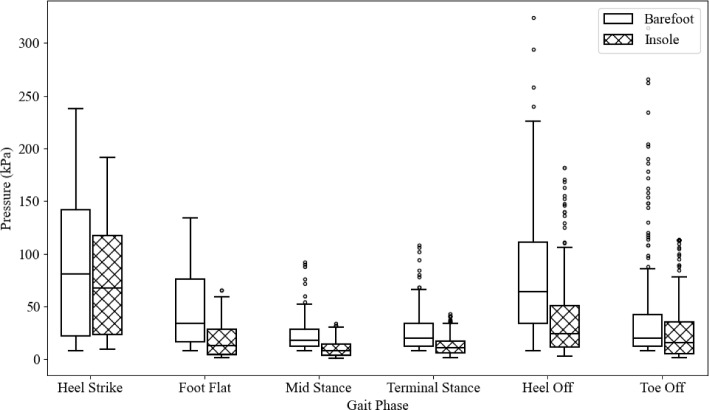
Experimental plantar pressures through gait for barefoot and insole wearing cases

Figure 34 shows the pressure values through each stage of gait for the barefoot and insole-wearing results. No filter was applied before processing these results.

Significant differences exist between the simulated plantar pressures with the optimised insole and the experimental data, particularly in the heel strike stage. The experimental pressures in this stage are significantly higher. This may be because the simulated pressure shows the arch is already taking some pressure, whereas the experimental results show just the heel taking the majority of the load. This difference may be due to inaccuracies in the modelling process with the contact angle or assumptions made in the contact definition. It could also be explained by natural variations in gait.

The experimental results also show lower peak pressures during the foot flat, mid stance and terminal stance stages. This may be due to assumptions made in the modelling with the contact and geometry of the bone structure. The contact was defined so no separation was allowed after the surface was in contact. This may have restricted the relative movement between the foot and the insole, introducing shear stresses that could have increased the plantar pressure. Additionally, the bone structure had several inaccuracies due to the non-subject-specific geometry and modifications made to align them within the bulk soft tissue. This created pressure points that were not aligned with experimental results.

Another notable difference is the pressure difference between the heel off and toe off stages. In previous modelling and experimental analysis, the toe off stage had higher peak pressures than the heel off stage due to the reduced contact area and angle of contact at the first toe. The primary reason for this may be because the forefoot bone structure in the numerical model had no movable joints, so the relative movement between the metatarsals and phalanges was not replicated in simulations. This would result in a significantly higher pressure at the first toe.

## Discussion

### Gait simulation numerical model

#### Static model

The static model aimed to verify the geometry and material properties of the foot geometry by simulating the subject standing. A comparison of numerical and experimental pressure measurements showed similar patterns with peak plantar pressures under the heel and metatarsal heads.

The main differences between the results may be due to the bone structure accuracy and representation of the soft tissues. It is suspected that more appropriate bone geometry would significantly improve the correlation between results. Due to limitations in this study, subject-specific bone geometry was not possible. Additionally, the material representation of the soft tissue may have led to further differences. The consecutive material model was taken from literature, however it should be noted that a large range of properties for soft tissue are available (Thomas et al. 2004; Chen et al. 2003; Erdemir et al. 2006; Chokhandre et al. 2012; Even-Tzur et al. 2006; Wang et al. 2014). Several sources show that many factors can play a role in the variability in plantar soft tissue properties such as age, health conditions and weight (Kwan et al. 2010; Pai and Ledoux 2010; Ledoux and Blevins 2007; Naemi et al. 2016). Chatzistergos et al. (2015) and Erdemir et al. (2006) emphasised that non-subject-specific properties can significantly alter plantar pressure predictions.

Despite the differences in the results, the overall plantar pressure pattern was deemed fit to continue using the generated geometry to further model the dynamic behaviour. However the inaccuracies caused by the simplifications made were carried through into further models.

Furthermore, it should be noted that the methods for obtaining the foot geometry were inefficient. Directly scanning the foot would typically be appropriate for creating a model. However, the subject was scanning their own foot in this circumstance, so it was the best method to obtain an accurate scan.

It should also be noted that the best approach for creating the foot geometry, specifically the bone structure, would be to segment the bones and tissue from MRI or CT images. Unfortunately this was not possible in this study due to budgetary constraints, which is why the alternative bone structure was used. A more appropriate demographic for the bone structure would have been advantageous. The bone adopted from Kathirgamanathan’s model were the closest available to this study at this time, and future studies should endeavour to obtain more appropriate geometry. The significant amount of scaling may have led to significant bone distortion, and the effects of using ill-fitting bone geometries should be investigated in future studies. Further work should also focus on creating a database of a range of bone geometries from a variety of demographics to avoid bone distortion. These demographics should include height, foot size, sex, and any abnormalities in foot structure.

#### Pressure-driven model

Although the results of the pressure-driven model appeared to align with those found in the literature, many approximations were made. The first is that the chosen frames accurately capture each stage of gait. This was done by comparing the pattern of peak pressures with existing results in the literature, but there was no quantitative analysis to confirm they were accurate. The next approximation was made when positioning the pressure maps. These maps were positioned to align with the regions of the foot that they appeared to correlate with, however minor errors may have been made in this process. The resulting reaction forces and moments at the proximal end of the ankle aligned well with trends seen in the literature, which provided confidence in the results.

#### Gait simulation model

Video analysis determined contact angles and rotational velocities at each gait stage. Selecting the frames for each gait stage was approximated by looking at the contacting regions and aligning them with the pressure results. A more accurate approach may have been to simultaneously film the contact angle while taking the plantar pressure measurements through gait. However, the results aligned with general patterns found in the literature.

Numerical results replicated experimental peak pressure locations well, although pressure concentrations differed. Numerical results generally showed greater variability and underestimated pressures in early gait phases (heel strike and foot flat). Experimental data showed higher median and quartile differences in early gait phases, possibly due to the numerical model’s inability to conform realistically to the ground or sensor damage during experiments. Despite the differences, the experimental and numerical results aligned well, both visually and quantitatively, particularly in the later stages of gait.

The numerical model has significant limitations based on the simplifications and assumptions made. These include lumping all soft tissue into a bulk soft tissue, the geometry of the bones and material models. All of these simplifications affect the mechanics of the foot and the way it is able to respond and conform to loading. Given the resources, having access to subject-specific bone geometry and constitutive material models is predicted to significantly improve the accuracy of the results. Additionally, including the mechanics of tendons, ligaments and the internal muscle forces would improve the gait simulation by providing the internal forces that facilitate foot mechanics while walking.

The limitations of a simplified model were balanced with the cost of running the optimisation process, both in time to repeat the initial model set up and the run time of the optimisation process. The lack of detail in the internal muscles, ligaments and tendons of the foot is speculated to have lowered the accuracy in the biomechanics of the foot. However, the results showed that the plantar pressure was replicated accurately. It was concluded that the simplified model is appropriate for this purpose, but the significance of this inaccuracy should be investigated in future studies.

The methodology in this paper could further be adapted for a wider range of people. The main focus of this study was focusing on improving foot comfort during walking, however the same methods could be used with different inputs for a variety of applications. Athletes or disabled people as a focus could follow the same methodology with varying plantar pressure inputs with their own personalised geometry.

### Optimisation

Static and dynamic numerical models compared the initialised lattice geometry with and without fluid. In both cases, the fluid had notable improvements. In the static model, these effects were minor quantitatively, where peak pressure was reduced by approximately 2%. However, comparing the pressure on the plantar surface between the two models showed more significant differences. The fluid-filled insole model shows a higher contacting area that is carrying load. This suggests that the addition of fluid may help imperfections in the total contact surface by inflating areas that have low or no pressure. There are more significant differences in the dynamic comparison between insoles with and without fluid, particularly in the later stages of gait. Comparing the pressure maps showed that the peak pressure under the first toe was more dispersed and had a lower magnitude. This may be because of the inflation effect that allows more area to carry load, lowering the peak pressure values. These comparisons suggest that even without modifying the internal structure of the insole, the addition of fluid improves the pressure distribution capabilities of an insole.

In general, the optimisation script performed well. It successfully reduced static peak pressures by approximately 8% and the summation of dynamic peak pressures by 50%. The improvement was much more pronounced in the dynamic model, suggesting that the customised material properties are more effective with dynamic loading. The biggest improvements in peak pressure were found in the later stages of gait where the pressures were much higher. The fluid appeared to improve the contact area, helping to offload the high pressure regions. Another notable benefit of the fluid is that it appeared to flow into imperfections in the insole where total contact was not achieved. This could be a beneficial outcome for insoles to compensate for inaccuracies in manufacturing to achieve better conformity to the plantar surface.

The offloading behaviour that lowered the peak pressures usually caused the midfoot region to carry more load. This created a more even pressure distribution, but did increase the load on the midfoot. It has been well reported in literature that a more even distribution of pressure, specifically that causes a higher midfoot load, is correlated with improved comfort (Che et al. 1994; Jordan et al. 1997; Murphy et al. 2005; Guldemond et al. 2007).

However, some issues were found that impeded the efficiency of the optimisation process. The most significant tissue was found when determining the direction vector for the next iteration. The vector would scale the changes in each parameter based on the change it had on the objective function when isolated. The issue when the changes combined to create a less favourable outcome. This was modified part way through the dynamic optimisation process, where only the most favourable changes were made rather than changing every parameter.

There was significantly less improvement in the static optimisation in comparison to the dynamic optimisation results. This may be because the insole geometry was designed to be in total contact with the plantar surface, so the initial properties already offered significant offloading. However, the peak pressures under the heel and first metatarsal were significantly reduced in the optimised geometry.

One of the more significant issues with this insole design process would be the time taken to create the final insole. In this initial study, the optimisation process took approximately 58 h, and the manufacturing of the insoles took approximately 12 h. Both of these processes would take approximately three days, however both of these processes are mostly without manual intervention. The time of production could be reduced by creating a more efficient optimisation process with a better initialisation point. This could be achieved by making typically high pressure regions such as under the heel, metatarsal heads, and large toe softer, and the lower pressure regions more stiff. This would allow the optimisation process to reach the final solution more efficiently.

Several assumptions made in the previous sections of the work amalgamated in the insole numerical model and optimisation process. One of the most significant assumptions that has not been previously discussed is the assumption that material properties for varying cell geometries can be interpolated linearly to define the constitutive material model in each region of the insole. As discussed in previous work (Cracknell 2024), the second-order hyperfoam can accurately model the response of the gyroid structure and shows consistency in the change in parameters with changes in geometry. Although this suggests good interpolation between the tested unit cell geometries, this assumption should be tested.

Another assumption that may have affected the results was that the numerical model of the insole did not capture the gradients between boundaries. Each insole region had a hard cut-off rather than a gradual change between regions. This did not appear to affect the result of the simulation, however future work should investigate whether implementing the gradient between regions would affect the optimisation or simulation results.

One of the major difficulties experienced in the experimental analysis was with the F-Scan sensor. The sensor was made from a stiff film that was difficult to place at the foot-insole interface and sit naturally with the topology. This may have significantly affected the results and was the primary reason the data had to be run through a Gaussian filter. The sensor would fold and crimp to fit the contours at the interface, interfering with the pressure readings. However, the effects were considered minor after the data was processed.

The results from the experimental test did not directly assess the effect of the fluid inside of the insole. The optimised insole was assessed as a whole, so it is unknown if the fluid had a significant effect in comparison to the structure. Future work should independently assess the structure and the SLC together for a direct comparison. Further studies should also look into the durability of the insole, including how the 3D printed material withstands pressure from the fluid as well as whether the material properties of the structure degrade in the long term.

## Conclusion

This paper presents a gradient-based optimisation method for creating dynamically customised SLC orthotic insoles. The findings suggest that fluid-filled insoles, regardless of optimisation, can improve plantar pressure distributions. The benefits for static loading are marginal, but significant when considering temporal pressure changes during gait. Optimised insoles provide further improvements, which are more pronounced with an optimised insole. Future studies should focus on improving the accuracy of bone geometry, investigating the durability of the insoles, and clinical studies on a diverse subject group.
